# Supervised retinal vessel segmentation from color fundus images based on matched filtering and AdaBoost classifier

**DOI:** 10.1371/journal.pone.0188939

**Published:** 2017-12-11

**Authors:** Nogol Memari, Abd Rahman Ramli, M. Iqbal Bin Saripan, Syamsiah Mashohor, Mehrdad Moghbel

**Affiliations:** Department of Computer & Communication Systems, Faculty of Engineering, University Putra Malaysia, Serdang, Selangor, Malaysia; Pennsylvania State Hershey College of Medicine, UNITED STATES

## Abstract

The structure and appearance of the blood vessel network in retinal fundus images is an essential part of diagnosing various problems associated with the eyes, such as diabetes and hypertension. In this paper, an automatic retinal vessel segmentation method utilizing matched filter techniques coupled with an AdaBoost classifier is proposed. The fundus image is enhanced using morphological operations, the contrast is increased using contrast limited adaptive histogram equalization (CLAHE) method and the inhomogeneity is corrected using Retinex approach. Then, the blood vessels are enhanced using a combination of B-COSFIRE and Frangi matched filters. From this preprocessed image, different statistical features are computed on a pixel-wise basis and used in an AdaBoost classifier to extract the blood vessel network inside the image. Finally, the segmented images are postprocessed to remove the misclassified pixels and regions. The proposed method was validated using publicly accessible Digital Retinal Images for Vessel Extraction (DRIVE), Structured Analysis of the Retina (STARE) and Child Heart and Health Study in England (CHASE_DB1) datasets commonly used for determining the accuracy of retinal vessel segmentation methods. The accuracy of the proposed segmentation method was comparable to other state of the art methods while being very close to the manual segmentation provided by the second human observer with an average accuracy of 0.972, 0.951 and 0.948 in DRIVE, STARE and CHASE_DB1 datasets, respectively.

## Introduction

Manual inspection of fundus images by a specialist known as a direct ophthalmoscopy is being challenged by the computer-assisted diagnosis of retinal images. Although direct ophthalmoscopy using retinal fundus images could be considered as an effective approach for diagnosing various retina related diseases that can result in blindness such as macular degeneration and diabetic retinopathy, it is time-consuming and the results cannot be easily reproduced. On the other hand, computer-assisted diagnosis of retinal fundus images has been shown to be as accurate as direct ophthalmoscopy while being faster and more reliable.

The condition and the appearance of the blood vessel network can be considered as an important aspect in many computer-assisted diagnosis systems using retinal fundus images. As a result, many techniques have been proposed for retinal blood vessel segmentation. However, it remains a challenging task due to the variations in vessel shape and width coupled with image acquisition difficulties that often results in a low-quality image with uneven illumination and a considerable amount of noise. Previously proposed retinal blood vessel segmentation methods can be categorized into supervised and unsupervised methods. Unsupervised approaches usually rely on different combinations of image processing concepts such as morphological operations, different filtering techniques and clustering methods, to name a few. On the other hand, supervised methods (mostly including machine learning based methods) utilize a set of pixel-wise features derived from the images to construct a set of rules that can be used for separating vessels and the background.

In this paper, retinal vessel segmentation methods are briefly discussed as it is intended to provide some insight to the overall segmentation concepts and is by no means an exhaustive review of these methods. For a detailed review of vessel segmentation methods please refer to [1–3]. Currently, thin vessels and the noise in retinal images can be considered as the main challenges in retinal vessel segmentation. Moreover, the majority of vessel segmentation methods optimize their preprocessing and segmentation steps for each dataset separately, resulting in high accuracy on specific datasets whereas the accuracy will suffer if applied to other datasets. Although there are some interactive and semi-automatic segmentation approaches, most of the vessel segmentation methods are considered as automatic. Usually, vessel segmentation approaches in retinal images include preprocessing steps aimed at enhancing the vessels, although some methods may skip this step and go directly to the segmentation.

Popular unsupervised retinal vessel segmentation methods can be divided into vessel tracking, matched filtering and morphology based methods. Starting from a set of initial points defined either manually or automatically, vessel tracking methods try to segment the vessels by tracking the center line of the vessels. This tracking can be done by utilizing different vessel estimation profiles such as Gaussian [4–6], generic parametric [7], Bayesian probabilistic [8] and multi-scale profiles [9]. On the other hand, filtering based techniques utilize different kernels for modeling and enhancing retinal vessels such as matched filters [10], Gaussian filters [11], wavelet filters [12, 13], Gabor filters [14, 15] and COSFIRE filters [16–18]. Methods utilizing morphological operations can be used for both the enhancement of retinal images and the segmentation of blood vessel tree from the background [19–21].

The supervised approach requires the ground truth segmentation provided by human experts (regarded as the gold standard) for training a classifier using a set of features calculated based on local (pixel-wise) or global image characteristics, acting as a priori knowledge and guiding the training. This set of features should be able to effectively discriminate between different objects of interest such as vessel and background pixels and can be extracted by different concepts such as Gabor filter responses and gray level co-occurrence matrices, to name a few. Various classification concepts can also be used to classify the pixels such as adaptive boosting (AdaBoost), support vector machines (SVM), artificial neural networks (ANN), Gaussian mixture models (GMM) and k-nearest neighbors (k-NN).

Niemeijer et al. [22] proposed a supervised retinal vessel segmentation where a k-NN classifier is utilized for identifying vessel and non-vessel pixels based on a feature vector constructed using a multi-scale Gaussian filter. Staal et al. [23] proposed a similar approach utilizing a feature vector constructed using a ridge detector where the ridge pixels are grouped into convex sets that approximately represent the straight lines. Based on a feature vector constructed using 2-D multi-scale Gabor wavelet filters. Marin et al. [24] proposed a feed-forward neural network (NN) based classifier utilizing 7-D feature vector calculated using moment-invariant features. Fraz et al. [25] proposed a classifier based on boosted decision trees using a 9-D feature vector computed from Gabor filter responses, morphological transformation, line strength measures and gradient vector field. Ricci et al. [26] proposed a fast and computationally non-demanding approach utilizing an SVM coupled with features derived using a rotation-invariant linear operator and pixel intensity.

Lupascu et al. [27] proposed an AdaBoost classifier using a 41-D feature set that could achieve good accuracy on DRIVE dataset. Wang et al. [28] proposed an ensemble based retina vessel segmentation method that is currently amongst the most accurate methods proposed. Their method is based on the creation of super-pixels using a simple linear iterative clustering (SLIC) approach where one pixel from each of the super-pixels is randomly selected for feature extraction. A trainable hierarchical feature extraction approach using a convolutional neural network (CNN) is then used on the selected pixel with an ensemble based Random Forest (RF) being used as the main classifier. You et al. [29] proposed an SVM based semi-supervised method utilizing features extracted using radial projection. Roychowdhury et al. [30] proposed a GMM classifier using 8-D features calculated from pixel neighborhood on first-order and second-order gradient images.

Zhu et al. [31] proposed an extreme learning machine (ELM) based segmentation using a 39-D feature vector constructed using morphological and local features coupled with features computed from phase congruency, Hessian and divergence of vector fields. Zhu et al. [32] proposed a similar approach using a classification and regression tree (CART) classifier using a 36-D feature vector constructed using multi-scale and multi-orientation morphological transformation and local features coupled with features computed from divergence of vector fields. Wang et al. [33] proposed an SVM based segmentation using a 30-D feature vector constructed using Gaussian and multi-scale Gabor filter features coupled with features computed from divergence of vector fields. Tang et al. [34] proposed an SVM based segmentation using a feature vector constructed using Multi-Scale vessel filtering and Gabor Wavelet features. Aslani et al. [35] proposed a random forest classifier based segmentation using a 17-D feature vector constructed using multi-scale and multi-orientation Gabor filter responses and intensity features coupled with features computed from vesselness measure and B-COSFIRE filter response.

Although most supervised retina vessel segmentation methods require an extensive and computationally demanding training phase, the results obtained are more accurate than unsupervised segmentation. In this paper, a supervised approach for automatic segmentation of retinal blood vessels in fundus images is proposed. The proposed method is based on an AdaBoost classifier coupled with the most informative pixel-wise features selected by a “minimal-redundancy-maximal-relevance” (mRMR) feature selection approach for increased accuracy and decreased computational requirements. The AdaBoost classifier is used as it has strong discriminative power and is computationally efficient. Moreover, a combination of filters is used to improve the segmentation as each filter responds in a distinct manner to different pixels in the image. By combining these filters, it is also possible to make the segmentation approach more robust considering the different image characteristics between datasets. Moreover, while many features related to pixels (such as intensity and texture) can be used, these features could have a high degree of redundancy with complicated interrelations leading to high computational requirements that make supervised segmentation resource intensive. As a result, feature selection has been used to reduce the computational requirements. Furthermore, the proposed method has not been optimized for any specific dataset as the goal of the study was to identify a set of optimal features and preprocessing parameters that could be used on a variety of datasets and images. The performance of the proposed method is validated using publicly accessible DRIVE [23], STARE [36] and CHASE_DB1 [37] datasets commonly utilized in assessing the performance of retinal vessel segmentation methods utilizing fundus images. REVIEWDB [38] and BioImLab [39] datasets were excluded as they are designed for use in methods dealing with vessel width and tortuosity estimation, respectively.

The rest of the paper is organized as follows: materials and methods section introduces the datasets used, the proposed image and vessel enhancement steps followed by segmentation steps including feature extraction and selection steps along with the AdaBoost classifier. In results and discussion section, the effects of feature selection and extraction parameters on segmentation accuracy is shown and the results of the proposed method and its performance is compared to recent methods from the literature. Finally, the conclusions are drawn.

## Materials and methods

**Fig 1:**
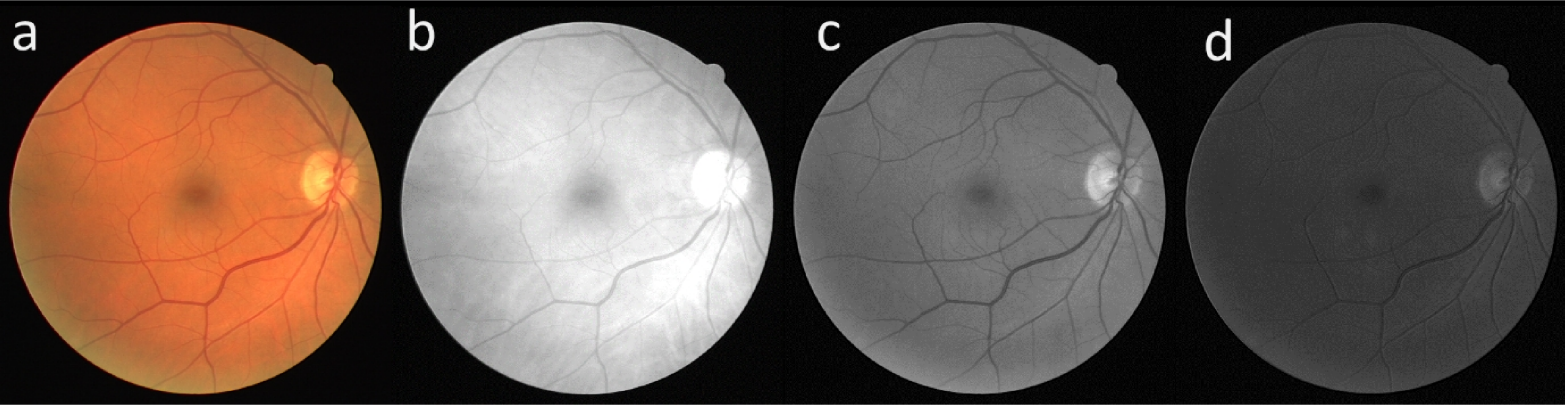
Color fundus image and its different RGB channels. (a) RGB image, (b) red channel, (c) green channel and (d) blue channel.

**Fig 2:**
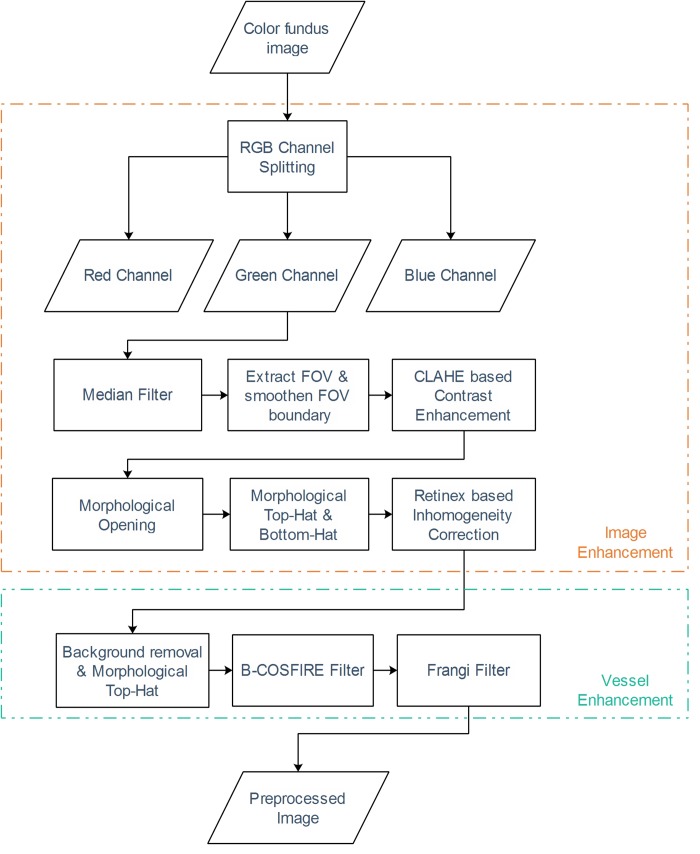
Preprocessing and vessel enhancement steps.

**Fig 3:**
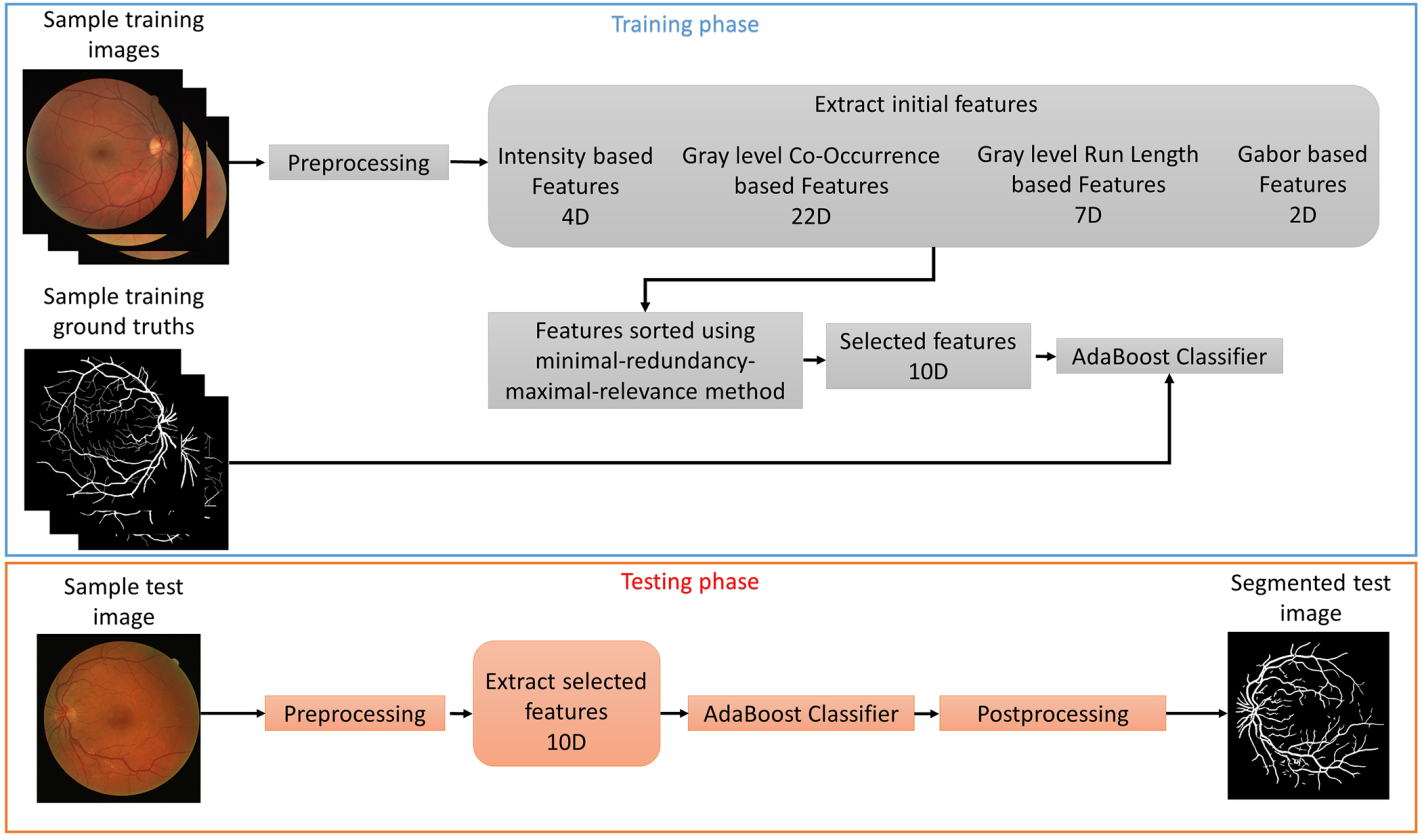
Feature extraction and classifier training/testing steps.

In this study, the green channel of the RGB fundus image is utilized per suggestions from many previous works as it was shown that vessels have the highest contrast against the background in the green channel. The use of blue channel results in a small dynamic range and red channel offers insufficient contrast, as illustrated in Fig 1. Moreover, Mendonca and Campilho [40] further validated the use of the green channel by comparing different channels of the RGB image, Luminance channel of National Television Systems Committee (NTSC) color space and *a* component of the *lab* image representation system where the green channel of the fundus image was shown to provide better contrast. Furthermore, like most other studies, only the pixels inside the FOV area of the image is considered for the segmentation as the pixels outside this area are considered as background and have no known medical applications. Fig 2 illustrates the proposed preprocessing and vessel enhancement steps and Fig 3 shows the feature extraction and classifier training/testing steps. These steps are discussed in detail in the following sections.

### Datasets

Datasets used in this study are amongst the most popular publicly accessible datasets used for development and testing the performance of various retinal segmentation methods. Datasets used also include corresponding vessel segmentation done manually by different experts considered as the ground truth.

**Fig 4:**
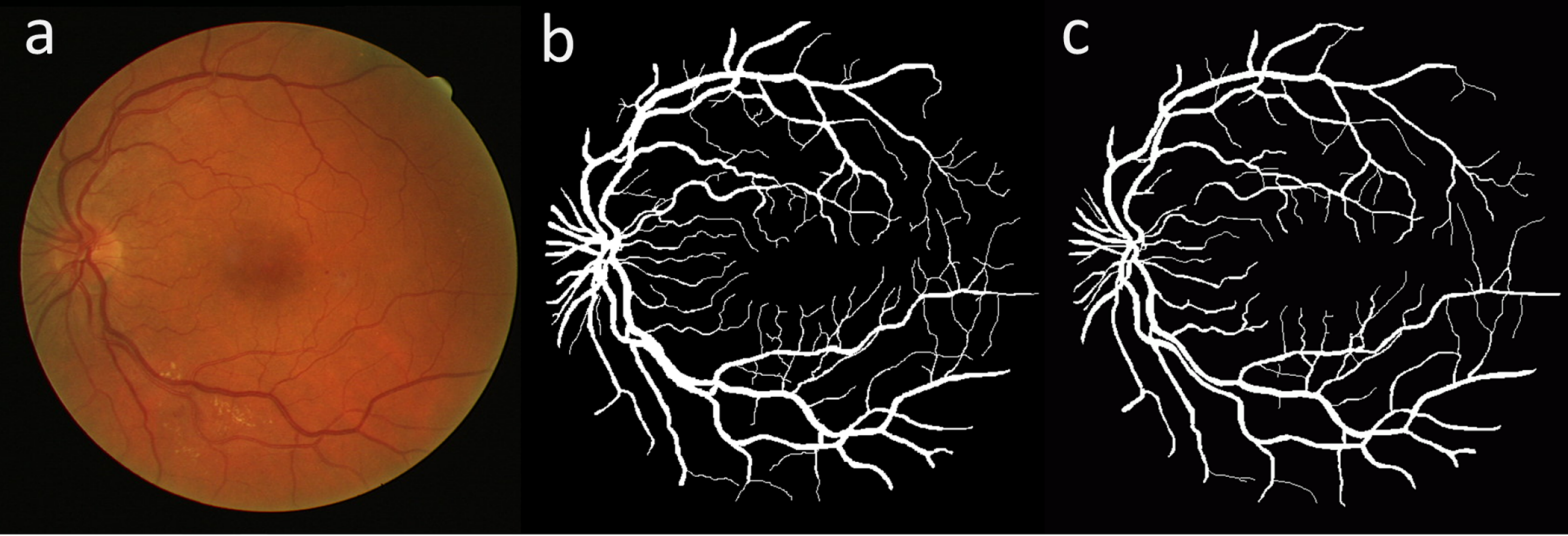
(a) An image from the DRIVE test set with its respective manual vessel segmentation, (b) first observer and (c) second observer.

DRIVE dataset includes 40 color fundus images divided equally into training and testing sets with each including 20 images [23]. For each image in the dataset, an FOV mask along with the manual segmentations of the corresponding vessel tree (one expert for training set and two experts for testing set) are provided. A Canon CR5 non-mydriatic camera with an FOV of 45° and bit depth of 8-bits was used to capture the images with a resolution of 768×584 pixels. Fig 4 illustrates an image from the test set of this dataset with its respective manual vessel segmentations.

**Fig 5:**
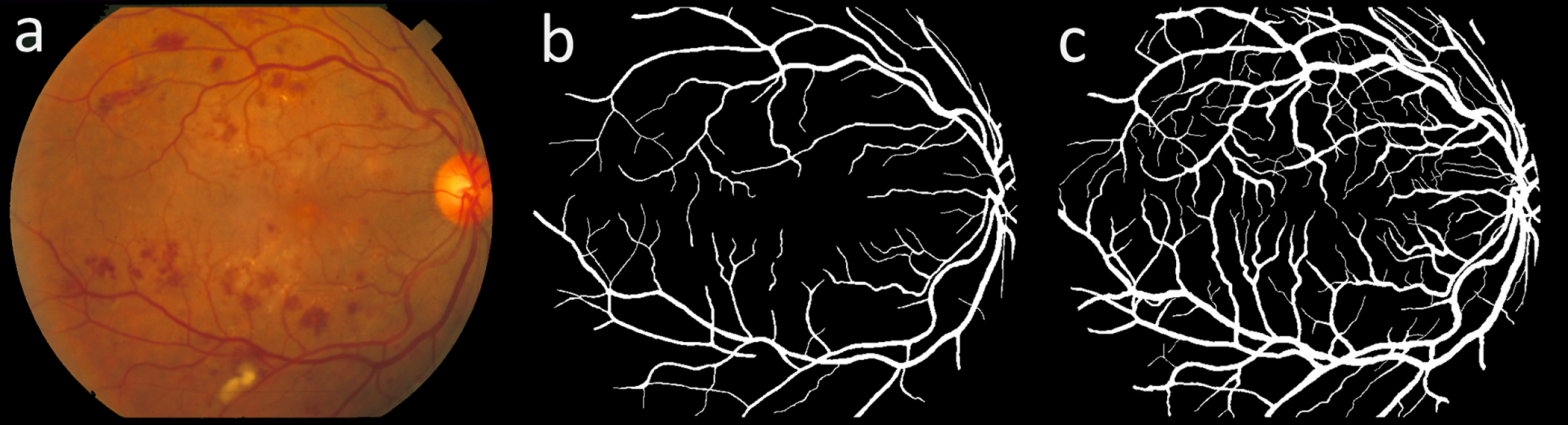
(a) An image from the STARE dataset with its respective manual vessel segmentation, (b) first observer and (c) second observer.

STARE dataset includes 20 color fundus images with half of them containing signs of different pathologies [36]. For each image in the dataset, manual segmentations of the corresponding vessel tree done by two experts are provided. A canon TopCon TRV-50 fundus camera with FOV of 35° and bit depth of 8-bits was used to capture the images with a resolution of 700×605 pixels. Fig 5 illustrates an image from this dataset with its respective manual vessel segmentations.

**Fig 6:**
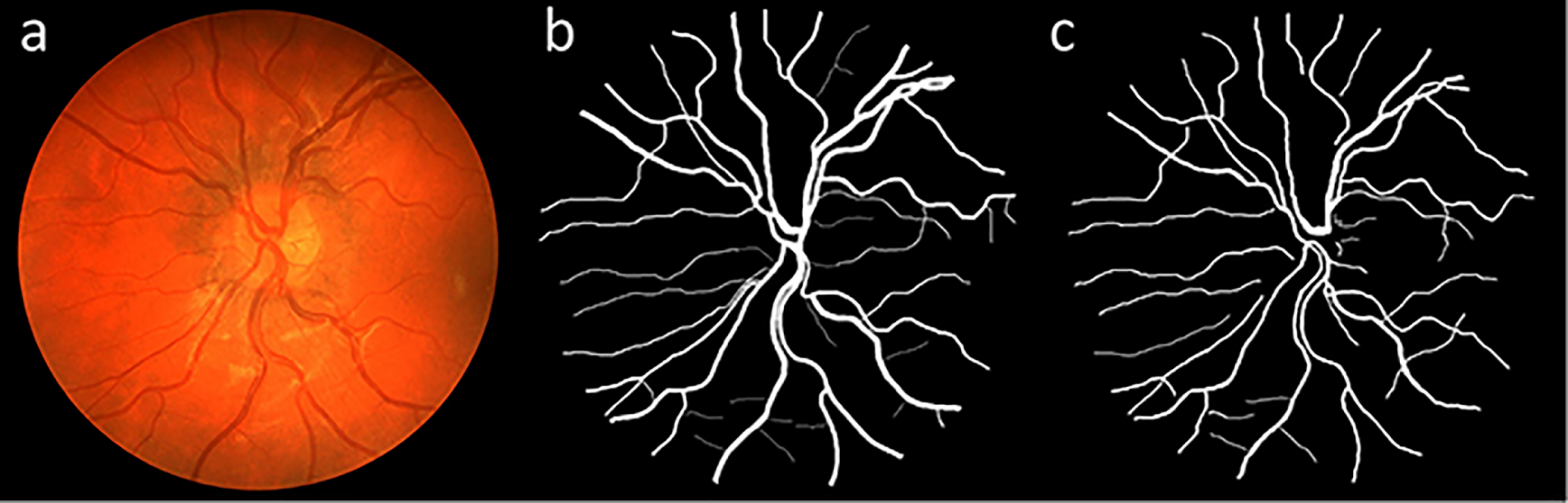
(a) An image from the CHASE_DB1 dataset with its respective manual vessel segmentation, (b) first observer and (c) second observer.

CHASE_DB1 dataset (referred to as CHASE in some publications) includes 28 color fundus images acquired from 14 patients participated in the child heart and health study in England [37]. For each image in the dataset, manual segmentations of the corresponding vessel tree done by two experts are provided. A Nidek NM 200D fundus camera with FOV of 30° and bit-depth of 8-bits was used to capture the images with a resolution of 1280×960 pixels. Fig 6 illustrates an image from this dataset with its respective manual vessel segmentations.

As seen from sample images, the manual segmentation provided by the first observer in DRIVE and CHASE_DB1 datasets includes finer segmentation for thinner vessels in contrast to STARE dataset where the manual segmentation provided by the second observer includes finer segmentation for thinner vessels.

### Preprocessing and vessel enhancement

The extracted green channel of the fundus image is first filtered using a 3×3 median filter for reducing the image noise as median filters have the useful property of retaining edge information within an image. As retina fundus images have high contrast around the edges of the image resulting in possible false positive vessels being detected around the edges of the image, the edges are smoothed [15, 17]. Initial FOV mask is computed by thresholding the luminance channel on *CIElab* color space [41] computed from the original RGB fundus image. Then, the FOV boundary is dilated by one pixel where the value of the new pixel is calculated as the mean value of its 8-connected neighbor pixels. This process is repeated fifty times for ensuring that no false vessels will be detected near the FOV boundary [17].

Contrast limited adaptive histogram equalization (CLAHE) algorithm [42, 43] is then used for enhancing the contrast between vessels and the background. CLAHE improves the local contrast and does not over amplify the noise present in relatively homogeneous regions. Then, the noise is further reduced by having the images morphologically opened. Finally, the image is further enhanced using a combination of morphological Top-hat and Bottom-hat operations.

#### Retinex based inhomogeneity correction

Often, retinal fundus images can include illumination inhomogeneity resulting in reduced segmentation accuracy. As a result, an inhomogeneity correction step is highly desired with Retinex theorem proposed by Land and McCann [44] being one of the most popular approaches. Retinex theorem, named after a combination of words retina and cortex, is used to remove the illumination inhomogeneity from the images, improving the segmentation accuracy [45–48]. In this study, a bilateral filter based Retinex algorithm is used as this approach was shown to provide better results in retinal images while preserving the vessels edge details [49, 50]. Based on Retinex theorem, any given image *I* can be represented as a component-wise multiplication of its reflectance *R* and illumination *L* as:

I=R×L (1)

Let’s denote *x* as a pixel belonging to image *I*, the pixel *x* of the reflectance image *R*(*x*) could be obtained by computing the difference of the logarithms between the original image *I*(*x*) and the resulting image *L*(*x*) after applying a bilateral filter to the original image *I*(*x*), defined as:

R(x)=log⁡(I(x)+1)−log⁡(L(x)+1) (2)

$$L(x)=M^{-1}(x)\int_{w}I(l)g(l,x)s(l,x)dl$$ (3)

$$M(x)=\int_{w}g(l,x)s(l,x)dl$$ (4)

$$g(l,x)=e^{-\frac{1}{2}{\left(\frac{d(l,x)}{\sigma_{d}}\right)}^{2}}$$ (5)

$$s(l,x)=e^{-\frac{1}{2}{\left(\frac{d(I(l),I(x))}{\sigma_{r}}\right)}^{2}}$$ (6)

Where *w* represents the window size used for measuring the spatial relations between pixels with a window size of 3×3 pixels being used in this study as suggested by [46] and *M* is a normalization factor. g(l,x) represents the spatial closeness (computed using Euclidean distance) between pixels *x* and l inside the window (*w*) and s(l,x) represents the intensity similarity between these pixel pairs. *σ*_*d*_ represents the spatial spread (based on low-pass filtering) and *σ*_*r*_ represents the spread of the image intensity range with *σ*_*r*_ and *σ*_*d*_ value of 0.3 used in this study. Afterwards, the image background is computed and removed by subtracting the image from its median filtered image using a large kernel.

#### B-COSFIRE filter

Bar-selective combination of shifted filter responses (B-COSFIRE) was proposed by Azzopardi et al. [17, 51] for detection of patterns with a bar shape. Vessels are enhanced with B-COSFIRE filter by using a bank of collinearly aligned Difference of Gaussian (DoG) filters that have been configured for detecting the bar-like appearance of blood vessels at different angles. A DoG filter for detecting the intensity variations of the image can be defined as:

$$DoG(x,y)=\frac{1}{2\pi \sigma^{2}}exp(-\frac{x^{2}+y^{2}}{2\sigma^{2}})-\frac{1}{2\pi {\left(0.5\sigma \right)}^{2}}exp(-\frac{x^{2}+y^{2}}{2{\left(0.5\sigma \right)}^{2}})$$ (7)

**Fig 7:**
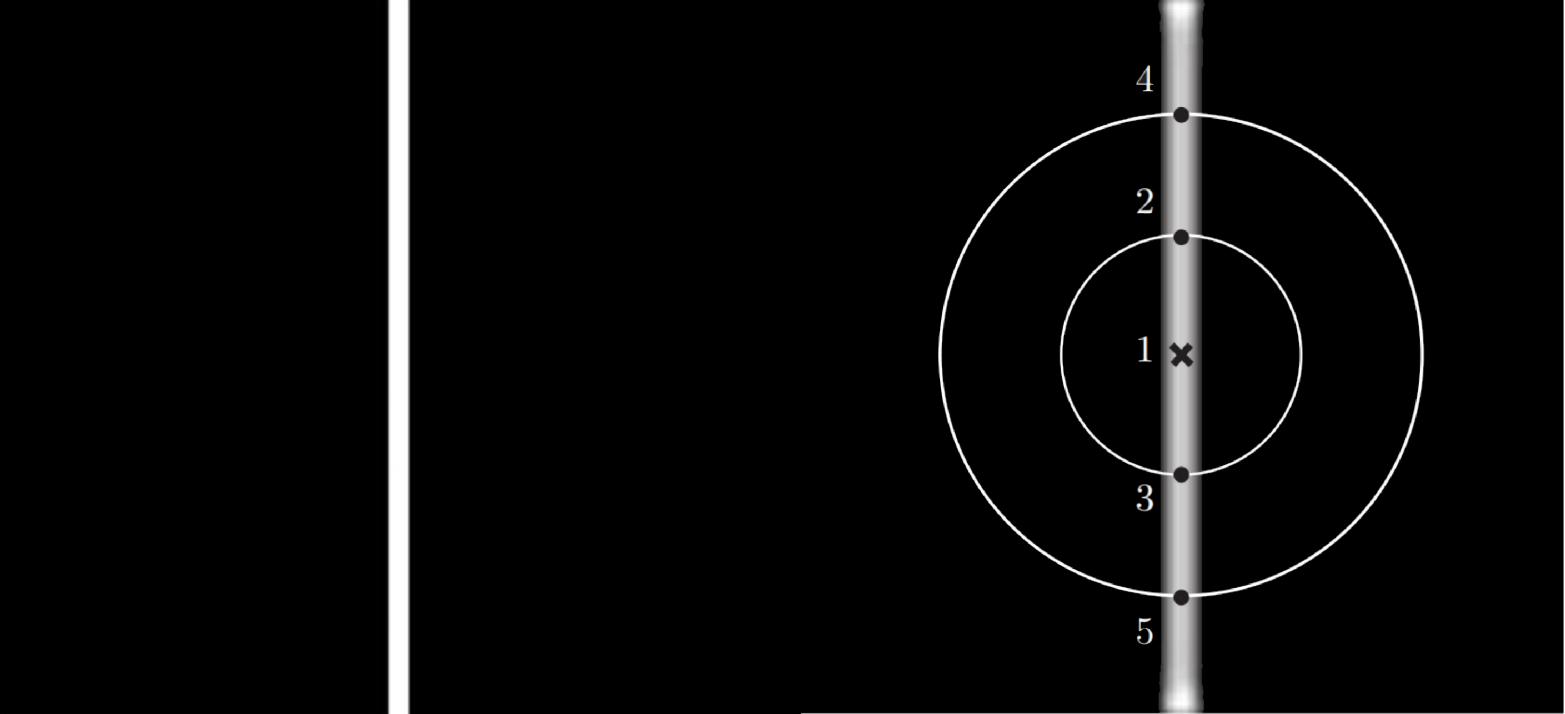
An example of symmetric B-COSFIRE filter configured to detect vertical bars with the support center being indicated by the cross marker [17]. The numbered dots along the concentric circles represent the positions with the strongest DoG responses.

Where *σ* denotes the standard deviation of the Gaussian function. The response *c*_*σ*_(*x*,*y*) of a DoG filter is computed by convolving image *I (x*,*y)* with *DoG (x*,*y)* while replacing any negative values with zero. Fig 7 illustrates a B-COSFIRE filter configured for detecting a vertical bar. Let’s consider point ‘1’ as the center point for a set of concentric circles and responses *c*_*σ*_(*x*,*y*) along these circles, points ‘1’ till ‘5’ represent significant responses to significant intensity changes (assuming a circle with radios of zero at the center point). These points are represented using set *S* defined as:

$${S=\{(\rho }_{i},\Phi_{i})|i=1,\ldots n\}$$ (8)

Where *ρ*_*i*_ and *Φ*_*i*_ represent the polar coordinates and *n* represents the number of DoG filter responses being considered. For the example image shown in Fig 7, *S* = {(0,0)_1_, (2,*π*/2)_2_, (2,3*π*/2)_3_, (4,*π*/2)_4_, (4,3*π*/2)_5_} with subscripts denoting the points with their position in the set *S*. By using this configuration, a B-COSFIRE filter is configured that is selective for the collinear alignment of significant intensity changes in an image (such as vessel patterns). By utilizing the DoG filter responses from different positions in set *S*, the B-COSFIRE filter output at the center points is determined. First, the DoG filter responses are blurred for allowing some tolerance in the position of the points being considered. The blurring is done by keeping the maximum value of the weighted DoG filter responses where a Gaussian function *G*_*σ*′_(*x*,*y*) is multiplied with DoG filter responses for the weighting. The standard deviation *σ*′ is considered as a linear function defined as *σ*′ = *σ*_0_ + *αρ*_*i*_ where *σ*_0_ and *α* are constants. Then, the responses are centered at the center point of DoG filter where each blurred DoG filter response is shifted by distance *ρ*_*i*_ with an opposite direction to *Φ*_*i*_. The DoG filter response $s_{\rho_{i},\Phi_{i}}(x,y)$ at each position (*ρ*_*i*_,*Φ*_*i*_) is computed as:

$$s_{\rho_{i},\Phi_{i}}(x,y)=\underset{x^{\prime },y^{\prime }}{\underbrace{max}}\;\{c_{\sigma }(x-\Delta x_{i}-x^{\prime }-\Delta y_{i}-y^{\prime })G_{\sigma^{\prime }}(x^{\prime },y^{\prime })\}$$ (9)

Where:

$${-3\sigma }^{\prime }\leq x^{\prime },y^{\prime }\leq {3\sigma }^{\prime }$$ (10)

Shift values are given as:

$$\Delta x_{i}=-\rho_{i}\cos\Phi_{i},\;\Delta y_{i}=-\rho_{i}\sin\Phi_{i}$$ (11)

The output of the B-COSFIRE filter is then defined as the weighted geometric mean of all the blurred and shifted responses from the set *S* as:

$$r_{s}(x,y)={\left|{\left({\prod_{i=1}^{n}\left(s_{\rho_{i},\Phi_{i}}(x,y)\right)}^{\omega_{i}}\right)}^{\frac{1}{\sum_{i=1}^{n}}\omega_{i}}\right|}_{t}$$ (12)

$$\omega_{i}=\exp\left(-\frac{\rho_{i}^{2}}{2{\hat{\sigma }}^{2}}\right)$$ (13)

$$\hat{\sigma }=\frac{1}{3}\;\underset{i\epsilon \{1,..,n\}}{\underbrace{max}}\{\rho_{i}\}$$ (14)

Where |.|_*t*_ denotes the thresholding of the responses at a fraction of their maximum value where *t* = (0 ≤ *t* ≥ 1). It should be noted that a B-COSFIRE filter will have a response only in case of non-zero values from $s_{\rho_{i},\Phi_{i}}(x,y)$ as the weighted geometric mean is an ‘AND’ type function. By moving further from the center point of the B-COSFIRE filter, the contribution of the blurred and shifted responses decrease.

The orientation of the bar structures used in the configuration determines the orientation preference of a B-COSFIRE filter. As such, it is possible to achieve rotation invariance by considering outputs *r*_*s*_(*x*,*y*) computed from a set of bar structures oriented at angles ranging from 0 to π. Bar structures oriented at angle *θ*_*k*_ can be computed by considering a new set $R_{\theta_{k}}(S)$ generated with respect to *S* as:

$$R_{\theta_{k}}(S)=\{(\rho_{i},\phi_{i}+\theta_{k})|\forall (\rho_{i},\phi_{i})\in S\}$$ (15)

Essentially, the process for obtaining responses *r*_*s*_(*x*,*y*) is the same as *s*(*x*,*y*) where only the bar orientations differ. In practice, twelve angles with equal intervals are used such as *θ*_*k*_ = {*kπ*/12 | 0 ≤ *k* ≤ 12} where the maximum response value of the B-COSFIRE filters with different orientations is taken at every location (*x*,*y*) as:

$${\hat{r}}_{s}(x,y)=\max_{\theta \in \phi }\left\{r_{R_{\theta_{k}}(S)}(x,y)\right\}$$ (16)

**Fig 8:**
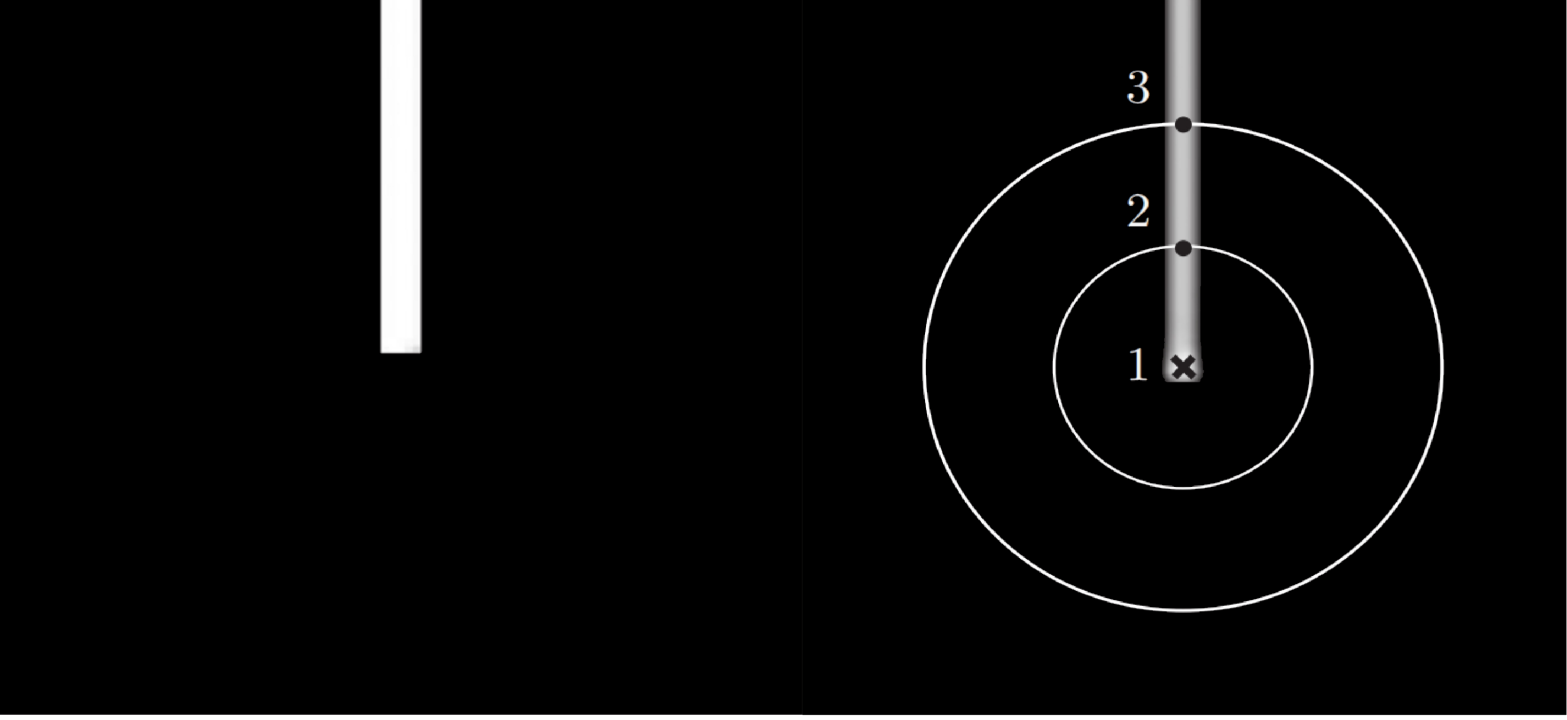
An example of asymmetric B-COSFIRE filter configured to detect vertical bar endings with the support center being indicated by the cross marker [17]. The numbered dots along the concentric circles represent the positions with the strongest DoG responses.

Due to ‘AND’ type output function of a B-COSFIRE filter, there should be no filter response at the end of the bars. However, due to the noise present in the images, a response will be computed although with a much lower value compared to a middle point in the bar. As such, a new B-COSFIRE filter was introduced that is selective for the bar ending known as asymmetric B-COSFIRE filter while the B-COSFIRE filter previously described is known as symmetric B-COSFIRE filter [17]. By placing the center point of the filter on the end point of the bar, a much higher response value is obtained. An example of symmetric and asymmetric B-COSFIRE filter responses can be seen in Figs 7 and 8, respectively. In this study, the responses from both the symmetric and asymmetric B-COSFIRE filters are summed together with the controlling parameters set empirically with *σ* = 2.4, *ρ* = {0,2,4,…,10}, *σ*_0_ = 3, *α* = 0.6 for symmetric and *σ* = 2.1, *ρ* = {0,2,4,…,18}, *σ*_0_ = 1, *α* = 0.1 for asymmetric filters, respectively.

#### Frangi filter

Frangi filters were first proposed for enhancing the vessel profiles in coronary artery segmentation [52]. Hessian matrix based Frangi filter is a popular approach that is both efficient and requires less computation time [53]. The Hessian matrix is constructed by computing the vertical and horizontal diagonals of the second-order derivative of the image. The Hessian based Frangi filter can be defined as:

$$F(x)={max}_{\sigma }f(x,\sigma )$$ (17)

Where the pixel of interest is defined by *x*, the standard deviation for computing the Gaussian derivative of the image is denoted as *σ* and *f* represents the filter. The hessian matrix can be defined as:

$$H=\left(\begin{array}{cc} H_{xx} & H_{xy}\\ H_{yx} & H_{yy} \end{array}\right)$$ (18)

Where *H*_*xx*_, *H*_*xy*_, *H*_*yx*_ and *H*_*yy*_ represent the directional second-order partial derivatives of the image. Let’s denote *λ*_*1*_ and *λ*_*2*_ as the eigenvalues of H, these are used for determining the probability of the pixel of interest *x* being a vessel based on the following notions:

$$|\lambda_{1}|\leq |\lambda_{2}|>0\;and\;f(x,\sigma )=0$$ (19)

Then, the Hessian based Frangi filter can be defined as:

$$f(x)=\left\{ \begin{array}{c} 0\;,\;if\;\lambda_{2}>o\\ e^{\left(-\frac{R_{b}^{2}}{2\;\alpha^{2}}\right)}\;(1-e^{\left(\frac{S^{2}}{2\beta^{2}}\right)}),\;elsewhere \end{array}\right.$$ (20)

$$R_{b}=\frac{\left|\lambda_{1}\right|}{\left|\lambda_{2}\right|},\;S=\sqrt{\lambda_{1}^{2}+\lambda_{2}^{2}}$$ (21)

**Fig 9:**
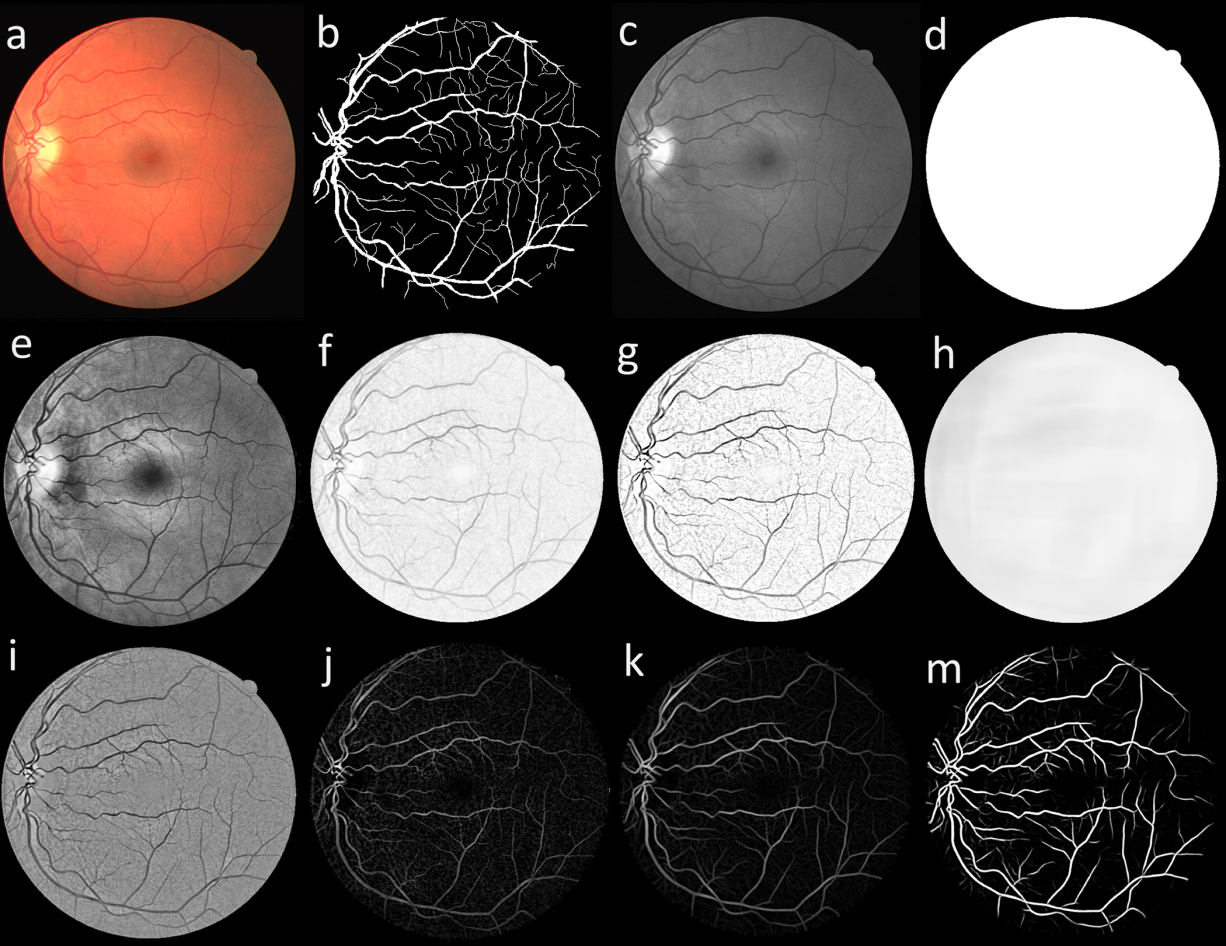
Preprocessing and vessel enhancement steps on an example image from DRIVE dataset. (a) color fundus image, (b) manual segmentation (first observer), (c) green channel, (d) FOV mask, (e) image after applying CLAHE, (f) image after applying Retinex, (g) image after applying morphological Top-hat and Bottom-hat, (h) computed background, (i) image after subtracting the background, (j) image after applying morphological Top-hat, (k) image after applying B-COSFIRE filter and (m) final preprocessed image after applying Frangi filter.

Where *R*_*b*_ and *a* are used for differentiating linear structures from blob-like structures while *β* and *S* are being used for differentiating background (noise) and vessels with the controlling parameters set empirically with *α* = 0.9, *β* = 13 and *σ* = {1,1.1,1.2,1.3,…,4}. Fig 9 illustrates the preprocessing and vessel enhancement steps and their effect on a sample image from DRIVE dataset.

### Extracting image features

The accuracy of machine learning based supervised segmentation approaches is highly dependent on the set of features being utilized. Thus, it is necessary to select the best set of features for a good separation between the vessels and the background. Sole use of intensity values as features is not that accurate and reliable and spatial relations between neighboring pixels should also be included in the features. In this study, a sliding window is used for extracting image features for each of the image pixels by considering a predefined set of pixels in the neighborhood of each pixel.

#### Intensity based image features

Intensity based features can be used to represent some texture characteristics of the image using the distribution of intensities inside the image. These features are mostly calculated using the image histogram. Given an image *I* with size *n*, these features are defined as:

$$Mean=\frac{1}{n^{2}}\underset{(i,j)\in {\left[1:n\right]}^{2}}{\sum }I_{i,j}$$ (22)

$$Variance=\frac{1}{n^{2}}\underset{(i,j)\in {\left[1:n\right]}^{2}}{\sum }(I_{i,j}-mean{)}^{2}$$ (23)

$$Skewness=\frac{1}{n^{2}}\underset{(i,j)\in {\left[1:n\right]}^{2}}{\sum }(I_{i,j}-mean{)}^{3}$$ (24)

$$Kurtosis=\frac{1}{n^{2}}\underset{(i,j)\in {\left[1:n\right]}^{2}}{\sum }(I_{i,j}-mean{)}^{4}$$ (25)

#### Image features calculated using gray level co-occurrence matrix

Co-occurrence matrices are used to track the distribution of the pixel pairs inside an image and are very popular in pattern recognition and image processing fields [54–56]. As this study uses gray intensity values for pixels, only one co-occurrence matrix will be used, known as gray level co-occurrence matrix (GLCM). The GLCM characterizes the image texture by calculating the adjacency occurrence of a pixel with specific intensity *i* to a pixel with intensity value *j* in a predefined distance *d* and angle *θ*. Although various distances can be used for calculating co-occurrence matrices, the choice of the angles is usually limited to angles as a multiple of 45° (0°, 45°, 90°, 135°), allowing direct use of intensity values without any interpolation (as co-occurrence matrices are rotationally invariant). To reduce dimensionality, for any given sliding window, a single GLCM from all four angles is calculated in this study. Let *P* define the GLCM of a quantized image *I* (*x*,*y*) with *P*(*i*,*j*) representing the GLCM and *N*_*g*_ representing the number of gray levels in image *I*. Only one GLCM of size *N*_*g*_ × *N*_*g*_ is computed per image per distance by simultaneously adding up the frequency of co-occurrences of all pixels with their connected neighbors with all pixels considered once as a center voxel. The entry (*i*,*j*) of the of the normalized GLCM is then defined as:

$$p\;(i,j)=\frac{P\;(i,j)}{\sum_{i=1}^{N_{g}}P\;(i,j)}$$ (26)

Before any discussion on image features computed using GLCM, the following notions should be considered:

$$p_{x}(i)=\sum_{j=1}^{L}p\;(i,j)$$ (27)

$$p_{y}(i)=\sum_{i=1}^{L}p\;(i,j)$$ (28)

$$\mu_{x}=\sum_{i}\sum_{j}i.p\;(i,j)$$ (29)

$$\mu_{y}=\sum_{i}\sum_{j}j.p\;(i,j)$$ (30)

$$\sigma_{x}^{2}=\sum_{i}\sum_{j}{\left(i-\mu_{x}\right)}^{2}.p\;(i,j)$$ (31)

$$\sigma_{y}^{2}=\sum_{i}\sum_{j}{\left(j-\mu_{y}\right)}^{2}.p\;(i,j)$$ (32)

$$p_{x+y}(k)={\sum_{i=1}^{L}}_{\;i+j=k}\sum_{j=1}^{L}p\;(i,j),k=2,3,\ldots ,2L$$ (33)

$$p_{x-y}(k)={\sum_{i=1}^{L}}_{\;|i-j|=k}\sum_{j=1}^{L}p\;(i,j),k=2,3,\ldots ,L-1$$ (34)

$$HX=-\sum_{i}p_{x}\;(i).\log\left(p_{x}\;(i)\right)$$ (35)

$$HY=-\sum_{i}p_{y}\;(i).\log\left(p_{y}\;(i)\right)$$ (36)

$$HXY=-\sum_{i}\sum_{j}p\;(i,j).\log\left(p\;(i,j)\right)$$ (37)

$$HXY1=-\sum_{i}\sum_{j}p\;(i,j).\log\left(p_{x}\;(i)p_{y}\;(j)\right)$$ (38)

$$HXY2=-\sum_{i}\sum_{j}p_{x}\;(i)p_{y}\;(j).\log\left(p_{x}(i)p_{y}(j)\right)$$ (39)

Based on above notions, GLCM features are defined as:

$$Autocorrelation=\sum_{i}\sum_{j}\left(i,j\right)p\;(i,j)$$ (40)

$$Energy=\sum_{i}\sum_{j}{p\;(i,j)}^{2}$$ (41)

$$Entropy=-\sum_{i}\sum_{j}p\;(i,j).\log\left(p\;(i,j)\right)$$ (42)

$$Correlation\;Ι=\sum_{i}\sum_{j}\frac{\left(i-\mu_{x})(j-\mu_{y})p\;(i,j\right)}{\sigma_{x}\sigma_{y}}$$ (43)

$$Correlation\;ΙΙ=\sum_{i}\sum_{j}\frac{(i.j)p\;(i,j)-\mu_{x}\mu_{y}}{\sigma_{x}\sigma_{y}}$$ (44)

$$Contrast\;=\sum_{i}\sum_{j}{\left|i,j\right|}^{2}p\;(i,j)$$ (45)

$$Cluster\;Shade\;=\sum_{i}\sum_{j}{\left(i+j-\mu_{x}-\mu_{y}\right)}^{3}p\;(i,j)$$ (46)

$$Cluster\;Prominence\;=\sum_{i}\sum_{j}{\left(i+j-\mu_{x}-\mu_{y}\right)}^{4}p\;(i,j)$$ (47)

$$Homogeneity\;Ι=\sum_{i}\sum_{j}\frac{p\;(i,j)}{1+|i-j|}$$ (48)

$$Homogeneity\;ΙΙ=\sum_{i}\sum_{j}\frac{p\;(i,j)}{1+{\left|i-j\right|}^{2}}$$ (49)

$$Inverse\;difference\;moment\;normalized=\sum_{i}\sum_{j}\frac{p\;(i,j)}{1+{\left(i-j\right)}^{2}/L}$$ (50)

$$Inverse\;difference\;normalized=\sum_{i}\sum_{j}\frac{p\;(i,j)}{1+{\left|i-j\right|}^{2}/L}$$ (51)

$$Maximum\;probability=\max_{i,j}p(i,j)$$ (52)

$$Sum\;average=\sum_{i=2}^{2L}i\;.\;p_{x+y}(i)$$ (53)

$$Sum\;variance=\sum_{i=2}^{2L}\;{\left(i+\sum_{i=2}^{2L}\;p_{x+y}(i).\log\left(p_{x+y}(i)\right)\right)}^{2}.p_{x+y}(i)$$ (54)

$$Dissimilarity=\sum_{i}\sum_{j}|i,j|.p\;(i,j)$$ (55)

$$Sum\;of\;squares:\;variance\;=\sum_{i}\sum_{j}{\left(i-v\right)}^{2}p\;(i,j)$$ (56)

$$Sum\;entropy=-\sum_{i=2}^{2L}\;p_{x+y}(i).\log\left(p_{x+y}(i)\right)$$ (57)

$$Difference\;variance\;=\sum_{i=0}^{L-1}\;i^{2}.p_{x+y}(i)$$ (58)

$$Difference\;entropy=-\sum_{i=0}^{L-1}\;p_{x-y}(i).\log\left(p_{x-y}(i)\right)$$ (59)

$$Information\;measure\;of\;correlation\;Ι\;=\frac{HXY-HXY1}{max(HX,HY)}$$ (60)

$$Information\;measure\;of\;correlation\;ΙΙ\;={\left(1-exp[-2(HXY2-HXY)]\right)}^{1/2}$$ (61)

#### Gray level run length matrix

Given an image, a gray level run can be defined as a set of consecutive pixels having the same gray level and being collinear in any given direction with the number of pixels in this set representing the length of the run. Gray level run length matrices (GLRLM) are used to represent this set where each element, denoted by *P(i*, *j*, *θ)*, contains the number of runs with length *j* with the gray level of *i* and the orientation *θ* representing line segment formed by the pixels [57, 58]. Gray level run length matrices can be computed by:

P(i,j,θ)=CARD[{(m,n)|f(m,n)=i,τ(i,θ)=j}] (62)

Where *f* (*x*, *n*) represents the gray level function for the pixel (*m*, *n*)_,_ and τ (*i*,θ) is the length of the gray level run *i* with direction *θ* and *CARD* denotes the cardinality of the set (number of elements). The choice of the angles is usually limited to angles as a multiple of 45° (0°, 45°, 90°, 135°) allowing direct use of intensity values without any interpolation (as GLRLM is rotationally invariant). To reduce dimensionality, for any given image a single GLRLM from all four angles is calculated. Utilizing the original run length matrix *P(i*, *j*, *θ)*, texture characteristics can be defined as:

$$Short\;Run\;Emphasis=\frac{1}{n_{r}}\;\sum_{i=1}^{M}\sum_{j=1}^{N}\frac{p\;(i,\;j)}{j^{2}}=\frac{1}{n_{r}}\;\sum_{j=1}^{N}\frac{p_{r}\;(i)}{j^{2}}$$ (63)

$$Long\;Run\;Emphasis=\frac{1}{n_{r}}\;\sum_{i=1}^{M}\sum_{j=1}^{N}p\;(i,j)\;.\;j^{2}=\frac{1}{n_{r}}\;\sum_{j=1}^{N}p_{r}\;(i)\;.\;j^{2}$$ (64)

$$Gray\;Level\;Non-Uniformity=\frac{1}{n_{r}}\;\sum_{i=1}^{M}{\left(\sum_{j=1}^{N}p\;(i,j)\right)}^{2}=\frac{1}{n_{r}}\;\sum_{i=1}^{M}p_{g}\;{\left(i\right)}^{2}$$ (65)

$$Run\;Percentage=\;\frac{n_{r}}{n_{p}}$$ (66)

$$Run\;Length\;Non-Uniformity=\;\frac{1}{n_{r}}\;\sum_{j=1}^{N}{\left(\sum_{i=1}^{M}p\;(i,j)\right)}^{2}=\;\frac{1}{n_{r}}\;\sum_{j=1}^{N}p_{r}\;{\left(i\right)}^{2}$$ (67)

$$Low\;Gray\;Level\;Run\;Emphasis=\;\frac{1}{n_{r}}\;\sum_{i=1}^{M}\sum_{j=1}^{N}\frac{p\;(i,j)}{i^{2}}=\;\frac{1}{n_{r}}\;\sum_{i=1}^{M}\frac{p_{g}\;(i)}{i^{2}}$$ (68)

$$High\;Gray\;Level\;Run\;Emphasis=\;\frac{1}{n_{r}}\;\sum_{i=1}^{M}\sum_{j=1}^{N}p\;(i,j)\;.\;i^{2}=\;\frac{1}{n_{r}}\;\sum_{i=1}^{M}p_{g}\;(i)\;.\;i^{2}$$ (69)

In above equations, *n*_*r*_ represents the total number of runs and *n*_*p*_ represents the total number of pixels in the image.

#### Using Gabor filters for texture characterization

For 2D images (spatial Gabor filter), convolution is used for applying Gabor filters where varying kernels are defined as Gaussian kernels modulated by a sinusoid [59, 60]. Using Cartesian basis as the center, these kernels are defined based on an abscissa with orientation *θ*. Gaussian and sinusoid components of the Gabor filter *K*_*θ*,*σ*,*γ*,*λ*,*φ*_ can be customized independently. Gabor filter *K*_*θ*,*σ*,*γ*,*λ*,*φ*_ is defined as:

$$K_{\theta ,\sigma ,\gamma ,\;\lambda ,\varphi }\;(x\;,y)=\exp\left(-\frac{x^{2}+\gamma^{2}y^{2}}{2\sigma^{2}}\right)\cos\left(2\pi \;\frac{x}{\lambda }+\varphi \right)$$ (70)

Where the Gaussian component is customized by its deviation *σ* and a spatial aspect ratio *γ* defining the ellipticity of the circular Gaussian and the sinusoid is customized by a spatial wavelength *λ* and a phase offset *ϕ*. The Gabor kernel is expressed using the orientation *θ* and with a change of scale defined by the size of pixel (*v*_*x*_,*v*_*y*_) and by a translation of the center of the kernel (*i*_*c*_,*j*_*c*_) as:

$$K_{\theta ,\sigma ,\gamma ,\;\lambda ,\varphi }\;(i\;,j)=\exp\left(-\frac{x^{\prime 2}+\gamma^{2}y^{\prime 2}}{2\sigma^{2}}\right)\cos\left(2\pi \;\frac{x^{\prime }}{\lambda }+\varphi \right)$$ (71)

Where:

$$x^{\prime }=(i-i_{c})v_{x}\cos\theta +(j-j_{c})v_{y}\sin\theta$$ (72)

$$y^{\prime }=(i-i_{c})v_{x}\sin\theta +(j-j_{c})v_{y}\cos\theta$$ (73)

A Gabor kernel is made of parallel stripes with different weights inside an ellipsoidal envelope with the parameters of the kernel controlling the size, the orientation and the position of these stripes. The wavelength *λ*, specified in pixels, represents the wavelength of the filter. This value is used for scaling the stripes whereas by modifying the wavelength, the overall size of the stripes is modified with the stripes keeping the same orientation and relative dimensions. This wavelength should be more than two and is often chosen to be less than the fifth of the image dimensions (because of the influence of image borders).

The angle of the parallel stripes is specified using the orientation *θ*. By modifying *θ*, the kernel can be rotated and oriented at the desired position. The shift of the cosine factor is represented by the phase offset *ϕ*. The symmetry of the kernel is determined by *ϕ* whereas by modifying the shift, positions of the inhibitory and excitatory stripes changes. The kernel is symmetric for a phase shift of *ϕ* = 0 and asymmetric for a phase shift of *ϕ* = *π*/4. Ellipticity is represented using the aspect ratio *γ*. The bandwidth *b* is used as a replacement for the Gaussian deviation *σ* [61, 62]. For extracting Gabor features, an image *R(x*,*y)* is defined where the input image *I(x*,*y*) is convolved with every Gabor filter *g(x*,*y)* from the banks of available filters as [63, 64]:

$$R(x,y)=g(x,y)*I(x,y)\sum_{m=0}^{M-1}\sum_{n=0}^{N-1}g(m,n)∙I(x-m,y-n)$$ (74)

Where * denotes 2D linear convolution and *M* and *N* are the size of the Gabor filter mask. The local squared energy *E(x*,*y)* of the filtered image can be obtained by computing the absolute mean deviation of the transformed values in the filtered images from the mean μ within a window *W* of size *M*_*x*_*M*_*y*_ as:

$$E(x,y)=(\frac{1}{M}\sum_{(a,b)\in W}\left|R(a,b)-\mu \right|{)}^{2}$$ (75)

Local responses from each one of the Gabor filters can also be represented in terms of amplitude *A(x*,*y)* defined as:

A(x,y)=|R(x,y)| (76)

To reduce dimensionality, for each scale *λ* a single mean value for squared energy and amplitude is calculated in this study.

#### Feature selection

As many of the extracted image features can be redundant or correlated, resulting in reduced performance in many classification methods, feature selection should be used for identifying the most prominent and informative features. Feature selection increases the classification accuracy by identifying the most discriminant features and their combination that might otherwise be hidden or contaminated by noisy features in a large feature vector. Another problem to consider during feature selection is the fact that the combinations of individually good features might not necessarily result in a good classification performance. In other words, “the *m* best features are not the best *m* features” [65]. It is likely that features selected using Max-Relevance criterion would have a high degree of redundancy, i.e. these features could have high dependency amongst them. This redundancy between features can result in a classifier with less than optimal performance. In this study, “minimal-redundancy-maximal-relevance” (mRMR) method proposed by [65] has been utilized for selecting the best set of features. The mRMR approach selects the most informative features using the maximal relevance criterion based on mutual information while minimizing the redundancy between the features and has gained wide popularity, especially in biomedical data analysis.

### AdaBoost classifier

Introduced by Freund and Schapire [66], Adaptive Boosting known as AdaBoost is a supervised machine learning technique that constructs a strong classifier by combining a set of low-level classifiers (known as weak learners) with low discrimination ability. The AdaBoost algorithm has become very popular in problems related to computer vision and medical imaging as it is simple to implement and is relatively fast. The AdaBoost algorithm can be easily applied to many problems without the need for extensive tuning. Only the number of training iterations needs to be defined, along with a set of weak learners. Moreover, the AdaBoost algorithm can quickly calculate the results of classification, this is especially useful in methods having lots of classifications. Given a training set, composed of features (samples) with ground truth classes, AdaBoost constructs a strong classifier by combining multiple weak learners in a coarse-to-fine approach. Although AdaBoost can be applied to multi-class classification problems, the context of this work only requires binary classification, therefore binary AdaBoost classification will be discussed. Given a training set *X* comprised of features and their classes (*x*_*i*_, *y*_*i*_), the goal of the AdaBoost algorithm is the construction of a strong classifier *H* that provides rules to predict the class of a sample *x*_*i*_ in the training set *X* with good accuracy. Given a set of weak learners ${\left\{h_{j}\right\}}_{j}$ and a maximum number of learning iterations *T*, the algorithm constructs this classifier *H* as a weighted sum of *t* weak learners balanced by their weights *αt*. It should be noted that a certain weak learner might be used more than once while some available weak learners might remain unutilized.

The AdaBoost learning process is based on defining a classifier that minimizes the prediction error for the training set *X*. This minimization problem could be considered as identifying a set of weights *a*_*i*_ along a set of weak learners {*h*_1_,…,*h*_*T*_} that minimize the classification error on the training set. As the expected class *y*_*i*_ is given for each sample *x*_*i*_, classification error is well defined. The main idea behind the learning process is to begin by classifying the easiest cases and then shifting the focus on more difficult cases. This process is based on a distribution D_*t*_ that assigns a weight to each training sample *x*_*i*_. During the learning process, the weights for well classified samples will decrease while the weights for misclassified samples will increase, thus shifting the focus on more difficult samples. The learning process is iterative, containing three main steps as shown in Algorithm 1.

**Algorithm 1. AdaBoost classifier**.

Given *X* = {(*x*_1_,*y*_1_),…,(*x*_*m*_,*y*_*m*_)} where *x*_*i*_ ∈ *X*, *y*_*i*_ ∈ *Y*, *x*_*i*_ and *y*_*i*_ ∈ {−1,+1}

**for** each training round t= {1, …, T}

        1.        Train a weak learner *h*_*t*_ using distribution *D*_*t*_

        2.        Choose $\alpha_{t}=\frac{1}{2}$ ln $\frac{1-\epsilon_{t}}{\epsilon_{t}}$ where $\epsilon_{t}=\sum_{i=1}^{m}D_{t}(i)[\left(h_{t}(x_{i})\neq y_{i}\right)]$

        3.        Update distribution as:

$$D_{t+1}(i)=\frac{D_{t}(i)}{Z_{t}}\times \left\{ \begin{array}{c} {exp}^{-\alpha_{t}}\;if\;h_{t}(x_{i})=y_{i}\;\\ {exp}^{\alpha_{t}}\;if\;h_{t}(x_{i})\neq y_{i} \end{array}\right.$$

**end**

Where $Z_{t}=2\sqrt{\epsilon_{t}(1-\epsilon_{t})}$

The final classifier is the defined as:

$$H(x)=sign\;\left(\sum_{t=1}^{T}\alpha_{t}h_{t}(x)\right)$$

First, a proper weak learner *h*_*t*_ should be chosen with an error smaller than 0.5 for the current distribution *D*_*t*_. Then the error ε_*t*_ is calculated with respect to the current distribution *D*_*t*_ and later used to define the weight *αt* for the weak learner *h*_*t*_. Finally, a new distribution *D*_*t*_+1 is calculated to focus more on samples that were not correctly classified during the step *t* while reducing the importance of correctly classified samples. A normalization term *Z*_*t*_ is also used so that *D*_*t*_+1 remains as a distribution. Often, samples are gives an equal weight at the initial distribution. However, in some cases such as in an unbalanced training set, the distribution might also be used to give some samples more weights. The algorithm stops when there is no error or when no weak learner has an error smaller than 0.5, as the classification accuracy cannot be improved on the training set. In the first instance, classification is already perfect in the training set, thus no further improvement is possible and in the second instance, the addition of more weak learners will not reduce the classification error. It should be noted that a tree-based week learner has been utilized in this study.

### Postprocessing

Due to the noise or anatomical structures that might be present in fundus images, there might be small blob-like regions of unconnected pixels wrongly identified as vessels (especially in pathological images). As a result, the final segmented image is cleaned by removing non-elongated and unconnected regions comprised of less than 30 pixels.

### Performance measures

#### Definitions of true and false positive/negative

- True positive: Vessel pixel correctly segmented as vessel pixel (TP)
- False positive: Non-vessel pixel segmented as vessel pixel (FP)
- True negative: Non-vessel pixel correctly segmented as non-vessel pixel (TN)
- False negative: Vessel pixel segmented as non-vessel pixel (FN)

#### Sensitivity

Sensitivity represents the probability that the segmentation method will correctly identify vessel pixels. Sensitivity is computed as:

Sensitivity=(totalTP)/(totalTP+totalFN) (77)

#### Specificity

Specificity is the probability that the segmentation method will correctly identify non-vessel pixels. Specificity is computed as:

Specificity=(totalTN)/(totalTN+totalFP) (78)

#### Accuracy

Accuracy represents the overall performance of a segmentation method. Accuracy is computed as:

Accuracy=(totalTP+totalTN)/(totalTP+totalFP+totalTN+totalFN) (79)

#### Area under a receiver operating characteristic curve

The area under a receiver operating characteristic curve (AUC), also known as AUROC, shows the performance of the segmentation method and is determined based on the trade-offs between sensitivity and specificity [67]. It should be noted that an AUC of 0.50 or less means that the segmentation algorithm is based purely on random guessing and an AUC of 1 means that the segmentation algorithm is able to segment all pixels correctly with respect to provided ground truth segmentation.

## Results and discussion

In this study, like most of the previous studies, the performance of the proposed vessel segmentation method is compared to the provided ground truth segmentations by the first observer (referred to as the first expert in some publications) in all datasets and the test set of the DRIVE dataset. While DRIVE dataset includes separate training and testing sets, training the classifier for use in STARE and CHASE_DB1 datasets is done differently as they do not have a separate training set. Currently, there are two popular approaches for training a classifier on STARE dataset. The first approach is based on the leave-one-out concept [15, 27] where one image is selected as test data and the classifier is trained using other images in the dataset with all the possible pixels used for the training, this process is repeated by changing the test image till all images in the dataset have been used once for testing. Second approach that is more popular (also used in this study) is based on randomly selecting a small number of pixels in each image and training the classifier using these samples with the use of 0.5% [26], 1% [35], 2% [28] and 6% [68] of the total pixels available in the dataset being suggested by different authors. In this study, a subset of 1% of randomly selected pixels inside the FOV from each image from STARE dataset and a subset of 5% of randomly selected pixels inside the FOV from each image from DRIVE training set is used for training the classifier as suggested by [35]. For CHASE_DB1 dataset, like the STARE dataset, a subset of 1% of randomly selected pixels inside the FOV from each image was used for training the classifier.

It should be noted that since the FOV masks are not provided in STARE and CHASE_DB1 datasets and to make the proposed method compatible with other datasets, FOV masks used were generated automatically and no dataset supplied FOV mask was used in this study. Table 1 illustrates the effects of different window sizes used for extracting image features on the classification accuracy using four randomly selected images from each of the datasets. Table 2 shows the accuracy implications of using different distances for calculating GLCM matrices computed on a 5×5 window utilizing an AdaBoost classifier with 200 learning cycles and 5-fold cross-validation using the same samples. As seen, the best segmentation accuracy can be achieved by using a window of 3×3 pixels and GLCM distance of one pixel.

**Table 1:** Effects of varying the feature extraction window size on classifier accuracy.

Window Size (pixels)	Sensitivity	Specificity	Accuracy
**3×3**	**0.812425**	**0.950528**	**0.932100**
5×5	0.804419	0.939632	0.922397
7×7	0.796018	0.915613	0.900553
9×9	0.814860	0.894425	0.884507
11×11	0.812207	0.871491	0.864167
13×13	0.807586	0.852919	0.847372
15×15	0.807140	0.832814	0.829701
17×17	0.810933	0.810378	0.810445
19×19	0.805950	0.790771	0.792581
21×21	0.797988	0.776182	0.778764
23×23	0.793468	0.761018	0.764832
25×25	0.779191	0.749188	0.752695

**Table 2:** Effects of varying GLCM distance on classifier accuracy computed on a 5×5 window.

GLCM Distance	Sensitivity	Specificity	Accuracy
**1**	**0.732950**	**0.867996**	**0.850784**
2	0.744058	0.840802	0.828467
3	0.709562	0.731677	0.728860
4	0.522021	0.843146	0.475131

**Fig 10:**
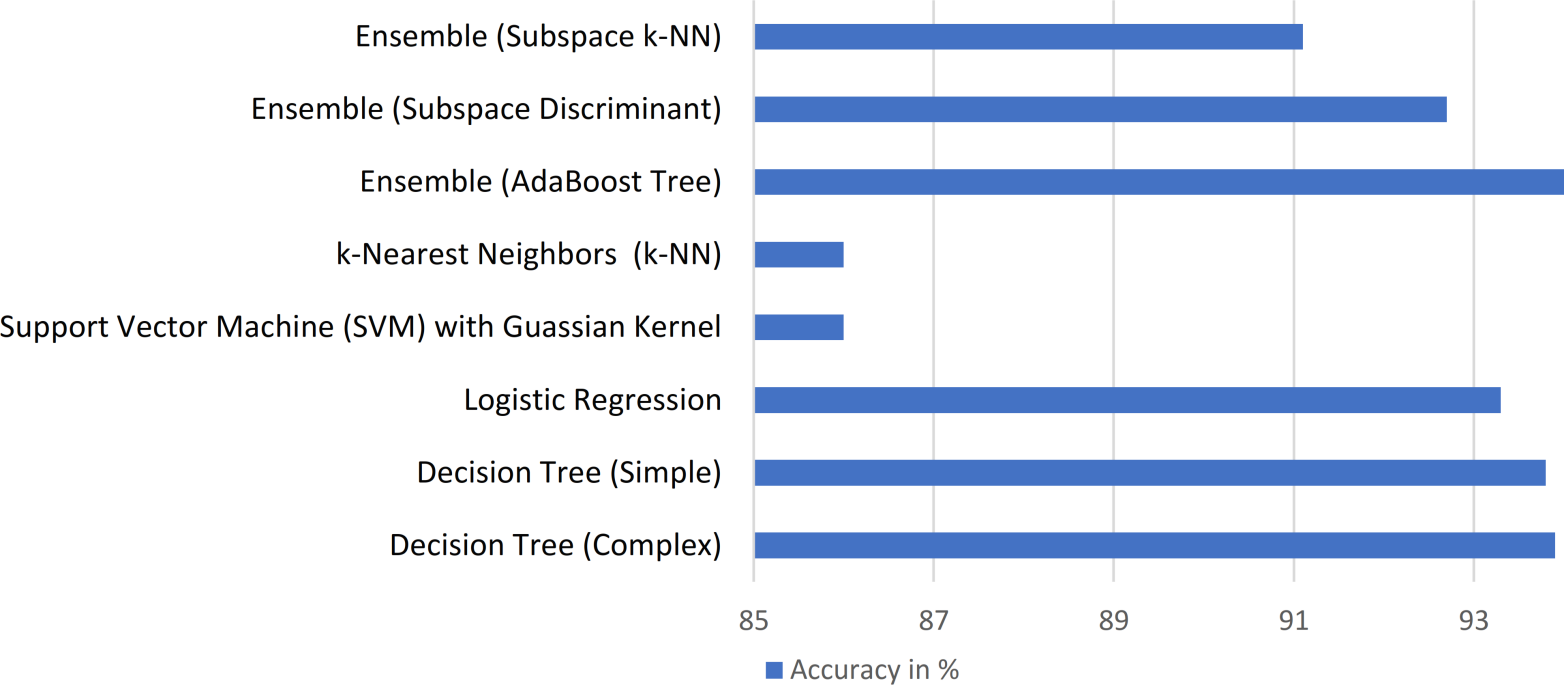
Retina vessel segmentation accuracy of different classifiers using 5-fold cross-validation on sample data.

The accuracy of different classification concepts was compared for selecting the most appropriate classification method for vessel segmentation using the same set of four randomly selected images from each of the datasets using 5-fold cross-validation with the AdaBoost classifier being the most accurate, as illustrated in Fig 10. As discussed, the accuracy of any classifier can be increased by selecting the most appropriate set of features and removing the redundant features. Table 3 illustrates different feature combinations sorted using the mRMR method and their effect on classifier accuracy using an AdaBoost classifier with 5-fold cross-validation. It should be noted that each feature combination contains all the features listed before that combination using the same set of four randomly selected images from each of the datasets. As seen, a combination of 10 features can result in the best overall classification accuracy while reducing the computational time by an average of 70.74%.

**Table 3:** Different combinations of features and their effect on vessel segmentation accuracy. It should be noted that each feature combination contains all the features listed before that combination.

Feature combinations	Features	Sensitivity	Specificity	Accuracy
1	Mean (intensity based)	0.773662	0.943587	0.919794
2	Cluster Prominence	0.853639	0.960726	0.945732
3	Run Length Non-Uniformity	0.863320	0.959784	0.946277
4	Gabor Mean Amplitude	0.858323	0.962049	0.947525
5	Low Gray Level Run Emphasis	0.858146	0.962143	0.947581
6	Difference Entropy	0.853626	0.963128	0.947795
7	Variance (intensity based)	0.854103	0.965016	0.949486
8	Sum of Squares: Variance	0.862384	0.959996	0.946328
9	Correlation II	0.853880	0.965101	0.949528
**10**	Gray Level Non-Uniformity	**0.849457**	**0.966444**	**0.950063**
11	Gabor Energy	0.874741	0.961355	0.949227
12	Contrast	0.852045	0.965521	0.949632
13	Energy	0.860752	0.964309	0.949809
14	Cluster Shade	0.871492	0.959857	0.947484
15	Homogeneity II	0.857430	0.961684	0.947086
16	Kurtosis	0.860113	0.960604	0.946533
17	Sum Variance	0.860113	0.960604	0.946533
18	Sum Entropy	0.849739	0.966287	0.949967
19	Information Measure of Correlation I	0.882341	0.958801	0.948095
20	Inverse Difference Normalized (INN)	0.874438	0.961006	0.948884
21	Skewness	0.868617	0.959980	0.947187
22	Information Measure of Correlation II	0.853262	0.962518	0.947220
23	Correlation I	0.870344	0.961700	0.948909
24	High Gray Level Run Emphasis	0.849505	0.965484	0.949244
25	Maximum Probability	0.870132	0.961736	0.948910
26	Sum Average	0.855573	0.962344	0.947394
27	Inverse Difference Moment Normalized	0.851663	0.964779	0.948940
28	Homogeneity I	0.856874	0.964978	0.949841
29	Long Run Emphasis	0.852692	0.964942	0.949224
30	Entropy	0.872157	0.959683	0.947427
31	Autocorrelation	0.851201	0.965210	0.949247
32	Dissimilarity	0.850687	0.965320	0.949269
33	Difference Entropy	0.850428	0.965386	0.949289
34	Short Run Emphasis	0.850653	0.965192	0.949154
35	Run Percentage	0.852630	0.965121	0.949370

**Fig 11:**
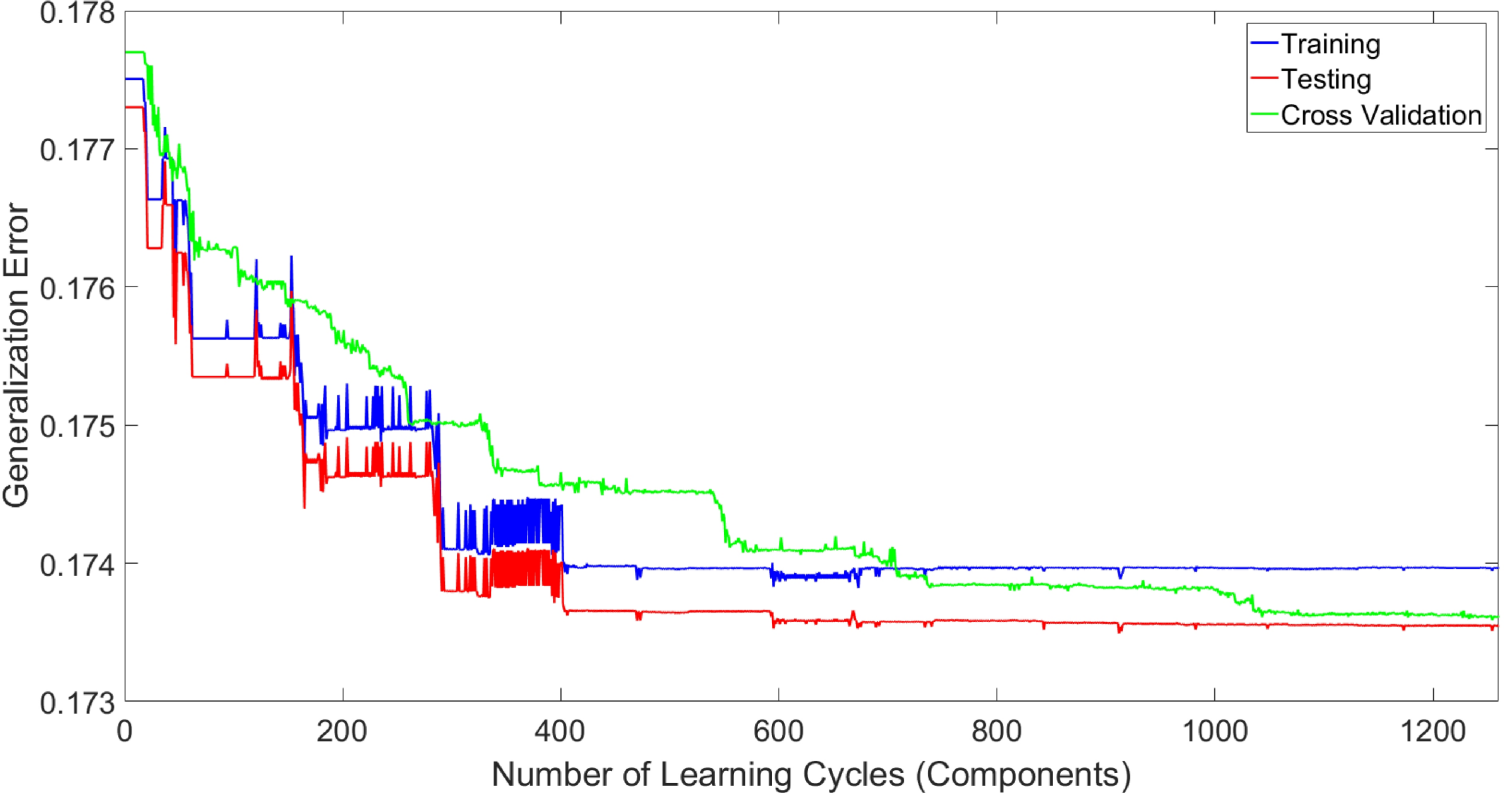
Generalization error for AdaBoost classifier using 5-fold cross-validation on sample data.

As mentioned before, the length (the number of components) of an AdaBoost classifier is determined by the maximum number of learning cycles given by the user during the learning process. As the AdaBoost method is based on a coarse-to-fine approach, adding components in later learning cycles of the training process can not only result in a low increase in the overall quality of classification, it can also increase the classification error. Thus, an optimal number of components (length) of an AdaBoost classifier should be determined using a validation step. This optimal length can be determined by finding the number of components of the classifier whereby adding more components, no improvement on generalization error can be seen. Fig 11 illustrates the generalization error for an AdaBoost classifier for vessel classification using 1,250 learning cycles using the same set of four randomly selected images using 5-fold cross-validation with 10 selected features per pixel (90% of samples were used for training and 10% were used for testing the classifier). As seen, by increasing the number of components the error decreases till 1100 components whereas the error begins to increase by adding more components. Based on validation results, training the classifier using 1080 learning cycles offers the best classification accuracy in this study.

This validation step offers many advantages. First, it improves the noise tolerance of the classifier. If some noise is present in the training set, these noisy samples are used in later stages of the learning process due to the coarse-to-fine approach of the AdaBoost classifier. Thus, by determining the optimal number of learning cycles, the influence of these noisy samples on overall classification function might be minimized. Then, the validation can be used for reducing classifier over-fitting by identifying the optimum number of learning cycles. Finally, the classification process will be faster as the classifier will not perform unnecessary computations. It should be noted that the proposed method was implemented in MATLAB R2016a using an Intel Core i7-3370 3.4GHz CPU coupled with 8 gigabytes of RAM with average classifier training time of 260 minutes per dataset.

Table 4 shows the performance of the proposed method compared to other state of the art segmentation methods for DRIVE and STARE datasets. Table 5 compares the performance of the proposed method and other state of the art segmentation methods that used CHASE_DB1 dataset. It should be mentioned that the first observer in DRIVE and CHASE_DB1 datasets has segmented thinner vessels compared to the second observer resulting in low sensitivity and high specificity while the second observer has segmented thinner vessels compared to the first observe in STARE dataset, resulting in high sensitivity and low specificity. Please note that the use of the character “-” in Tables imply that the performance metric was not implemented by the authors in their respective paper.

**Table 4:** A comparison between different retinal vessel segmentation methods evaluated using DRIVE and SATRE datasets.

Method	DRIVE				STARE			
	Sensitivity	Specificity	Accuracy	AUC	Sensitivity	Specificity	Accuracy	AUC
Second observer	0.7796	0.9717	0.9464	0.9466	0.8955	0.9382	0.9347	0.9686
**Unsupervised segmentation methods**								
Zhao et al. [69]	0.7354	0.9789	0.9477	-	0.7187	0.9767	0.9509	-
Azzopardi et al. [17]	0.7656	0.9704	0.9442	0.9614	0.7716	0.9701	0.9497	0.9563
You et al. [29]	0.7410	0.9751	0.9434	-	0.7260	0.9756	0.9497	-
Fraz et al. [70]	0.7152	0.9759	0.9430	-	0.7311	0.9680	0.9442	-
Lam et al. [71]	-	-	-	-	-	-	0.9567	0.9739
Al-Diri et al. [72]	0.7282	0.9551	-	-	0.7521	0.9681	-	-
BahadarKhan et al. [19]	0.746	0.980	0.961	0.863	0.758	0.963	0.946	0.861
Wang et al. [13]	-	-	0.9461	0.9543	-	-	0.9521	0.9682
Khan et al. [16]	0.7155	0.9805	0.9579	-	0.7728	0.9649	0.9518	-
Miri et al. [73]	0.7352	0.9795	0.9458	-	-	-	-	-
**Supervised segmentation methods**								
Staal et al. [23]	-	-	0.9441	0.9520	-	-	0.9516	0.9614
Soares et al. [15]	0.7332	0.9782	0.9461	0.9614	0.7207	0.9747	0.9479	0.9671
Lupascu et al. [27]	0.720	-	0.9597	0.9561	-	-	-	-
Marín et al. [24]	0.7067	0.9801	0.9452	0.9588	0.6944	0.9819	0.9526	0.9769
Wang et al. [13]	-	-	0.946	-	-	-	0.952	-
Ricci et al. [26]	-	-	0.9595	0.9633	-	-	0.9646	0.9680
Fraz et al. [25]	0.7406	0.9807	0.9480	0.9747	0.7548	0.9763	0.9534	0.9768
Cheng et al. [68]	0.7252	0.9798	0.9474	0.9648	0.7813	0.9843	0.9633	0.9844
Aslani et al. [35]	0.7545	0.9801	0.9513	0.9682	0.7556	0.9837	0.9605	0.9789
Niemeijer et al. [22]	-	-	0.9416	0.9294	-	-	-	-
Zhu et al. [31]	0.7140	0.9868	0.9607	0.9086	-	-	-	-
Zhu et al. [32]	0.7462	0.9838	0.9618	0.9419	-	-	-	-
Han et al. [74]	0.6770	0.9871	0.9473	-	0.7043	0.9869	0.9573	-
Wang et al. [28]	0.8173	0.9733	0.9767	0.9475	0.8104	0.9791	0.9813	0.9751
Peng et al. [75]	-	-	-	-	0.7256	0.9750	0.9492	-
Maharjan et al. [76]	0.6411	0.9625	0.9349	-	0.6162	0.9615	0.9353	-
Rodrigues et al. [77]	0.7654	0.9789	0.9607	-	0.6120	0.9787	0.9406	-
**Proposed Method**	**0.8726**	**0.9884**	**0.9722**	**0.9795**	**0.8085**	**0.9798**	**0.9514**	**0.9701**

**Table 5:** A comparison between different retinal vessel segmentation methods evaluated using CHASE_DB1 dataset.

Method	Sensitivity	Specificity	Accuracy	AUC
Second observer	0.767	0.985	0.969	0.9451
Fraz et al. [70] (supervised)	0.722	0.971	0.946	0.9712
Azzopardi et al. [17]	0.758	0.958	0.938	0.9487
Frangi et al. [52]	0.897	0.663	0.920	-
Chaudhuri et al. [5]	0.282	0.926	0.848	-
Chanwimaluang et al. [78]	0.508	0.943	0.913	-
Chakraborti et al. [79]	0.528	0.959	0.929	-
**Proposed Method**	**0.8192**	**0.9591**	**0.9482**	**0.9436**

The results obtained on the DRIVE dataset shows that the proposed method is amongst the top methods with an accuracy of 0.9722, sensitivity of 0.8726 and specificity of 0.9884. In the case of the results obtained from STARE dataset, with an accuracy of 0.9514, sensitivity of 0.8085 and specificity of 0.9798, the proposed method is amongst the top methods proposed for retinal vessel segmentation. Although the CHASE_DB1 dataset has received less interest from researchers, the proposed method was also amongst the top methods proposed and evaluated using CHASE_DB1 dataset with an accuracy of 0.9482, sensitivity of 0.8192 and specificity of 0.9591. The proposed method was able to achieve a higher AUC value compared to most other methods on DRIVE, STARE and CHASE_DB1 datasets with AUC of 0.9795, 0.9701 and 0.9436, respectively.

Moreover, the results show that the proposed method was more accurate than the segmentation provided by the second human observer for DRIVE and STARE datasets while being very close to the segmentation provided by the second human observer in CHASE_DB1 dataset. The results achieved are comparable to most supervised and unsupervised segmentation methods from the literature. The low interest for including CHASE_DB1 dataset in the development of vessel segmentation methods and lower segmentation accuracy observed in this dataset can be attributed to the non-uniform illumination in the background coupled with central vessel reflexes on some images and the low overall contrast between vessels, making accurate segmentation a challenging task. Although it is possible to increase the segmentation accuracy of the proposed method by adjusting the parameters for preprocessing and feature extraction/selection separately for each of the datasets, the goal of the study was to identify an optimal set of features, preprocessing and segmentation parameters that could be used on a variety of datasets and images.

**Fig 12:**
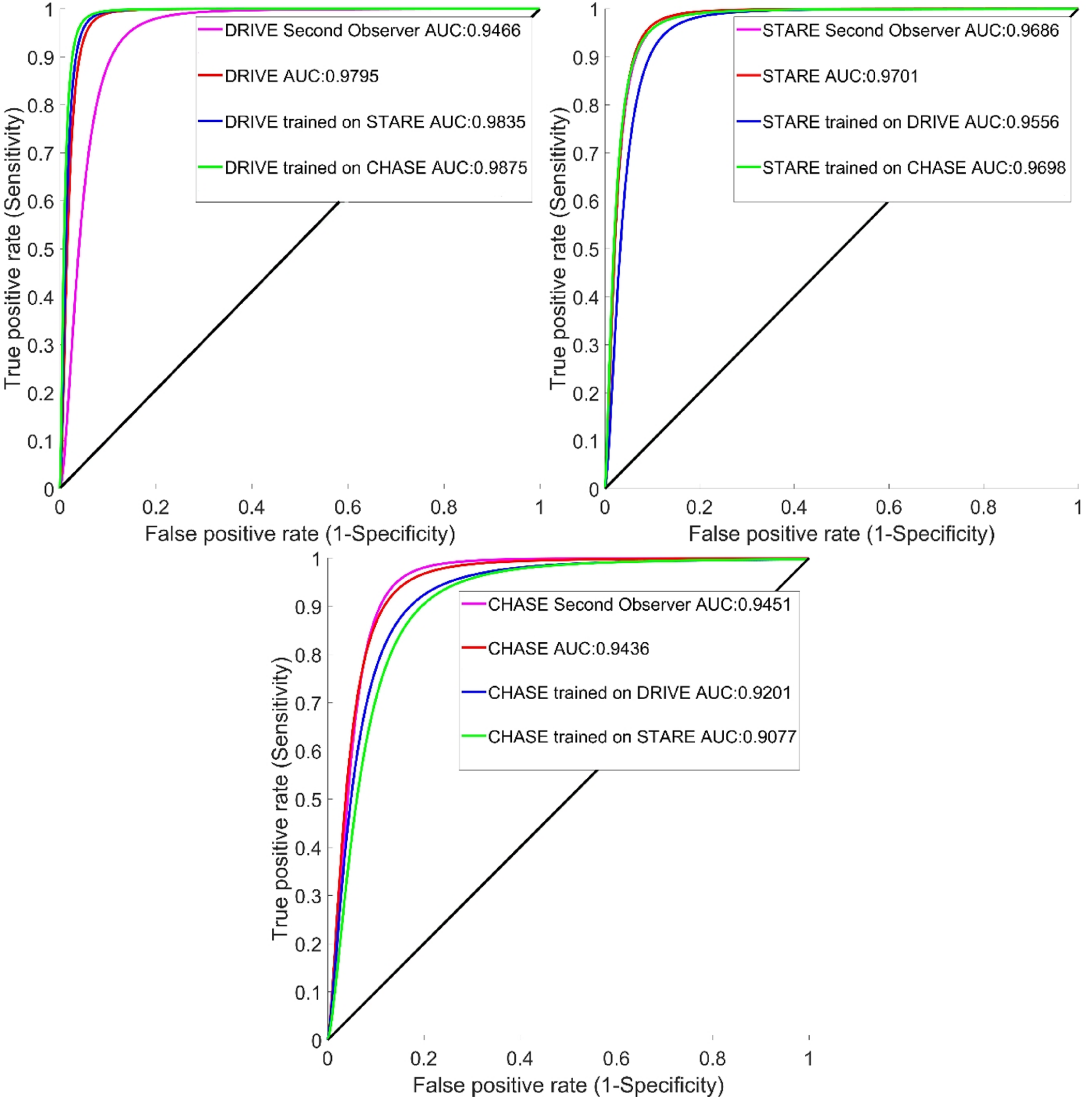
ROC curve of the proposed classifier. (a) DRIVE, (b) STARE and (c) CHASE_DB1 (CHASE) datasets.

For ensuring the robustness of supervised segmentation methods, a cross-training/testing approach is commonly used where a classifier is trained using one dataset and tested on other datasets and vice-versa. From a practical standpoint, this cross-training/testing can be a good measure of the effectiveness of supervised segmentation on unseen data with Table 6 illustrating the segmentation accuracy in case of this cross-training/testing. A total of 5%, 1% and 1% of the previously selected random pixels from DRIVE (train set), STARE and CHASE_DB1 datasets were used for training the classifiers, respectively. Table 7 compares the accuracy of different supervised methods proposed for retinal vessel segmentation in the case of cross-training/testing. As seen, the accuracy obtained for cross-training/testing is comparable to other methods from the literature with an accuracy of 0.9701 for DRIVE dataset trained using STARE dataset and an accuracy of 0.9484 for STARE dataset trained using DRIVE dataset. The proposed method can be considered independent of the training data as it did not show a considerable drop in accuracy compared to some other methods from the literature. The ROC curves of the proposed segmentation method for DRIVE, STARE and CHASE_DB1 datasets are illustrated in Fig 12. Table 8 shows the average segmentation time required by different vessel segmentation methods computed per image.

**Table 6:** The segmentation performance of the proposed method in case of cross-training/testing.

Dataset		Sensitivity	Specificity	Accuracy	AUC
**DRIVE**	Trained on STARE	0.8390	0.9915	0.9701	0.9835
	Trained on CHASE_DB1	0.7511	0.9949	0.9608	0.9875
**STARE**	Trained on DRIVE	0.8488	0.9682	0.9484	0.9541
	Trained on CHASE_DB1	0.7182	0.9862	0.9418	0.9698
**CHASE_DB1**	Trained on DRIVE	0.7735	0.9392	0.9224	0.9201
	Trained on STARE	0.7082	0.9556	0.9305	0.9077

**Table 7:** A comparison between the average accuracy of different segmentation methods with cross-training/testing.

Method	DRIVE Trained on STARE	STARE Trained on DRIVE
Soares et al. [15]	0.9397	0.9327
Ricci et al. [26]	0.9266	0.9452
Marín et al. [24]	0.9448	0.9526
Fraz et al. [25]	0.9456	0.9495
Cheng et al. [68]	0.9384	0.9476
Aslani et al. [35]	0.9496	0.9545
Wang et al. [28]	0.9803	0.9710
**Proposed Method**	**0.9701**	**0.9484**

**Table 8:** A comparison between the average processing time of different segmentation methods per image.

Method	Average processing time per image	Computational resources	Development environment
Khan et al. [16]	10.6 seconds	Intel Core i3 CPU running at 2.53 GHz, 4 GB RAM	MATLAB
BahadarKhan et al. [19]	1.5 seconds		MATLAB
Dai et al. [80]	1 minutes and 46 seconds		MATLAB
Zhao et al. [46]	4.6 seconds		MATLAB & C++
Mapayi et al. [81]	2.6 seconds	Intel Core i5 CPU running at 2.30GHz, 4GB RAM	MATLAB
Asad et al. [82]	2 minutes and 45 seconds	Intel Core i3 CPU running at 2.53 GHz, 3 GB RAM	MATLAB
Lam et al. [83]	13 minutes	Intel Core2duo CPU running at 1.83 GHz, 2 GB RAM	Not mentioned
Al-Diri et al. [72]	11 minutes	Intel Pentium 4 CPU running at 1.2 GHz	MATLAB
Staal et al. [23] (supervised)	15 minutes	Intel Pentium 4 CPU running at 1.0 GHz, 1 GB RAM	Not mentioned
Marín et al. [24] (supervised)	1.5 minutes	Intel Core2duo CPU running at 2.13 GHz, 2 GB RAM	Not mentioned
Fraz et al. [25] (supervised)	2 minutes	Intel Core i3 CPU running at 2.27 GHz, 4 GB RAM	MATLAB
Sofka and Stewart [84] (supervised)	2.3 seconds	Intel Core i5-M480 CPU running at 2.67 GHz, 4 GB RAM	C++
Soares et al. [15] (supervised)	18.7 seconds		MATLAB & C++
Bankhead et al. [12]	15 seconds		MATLAB & C++
Vlachos et al. [85]	6.5 seconds		MATLAB
Azzopardi et al. [17]	7.5 seconds		MATLAB & C++
Nguyen et al. [86]	4.6 seconds		MATLAB
**Proposed method**	**8.2 minutes**		**MATLAB**

**Fig 13:**
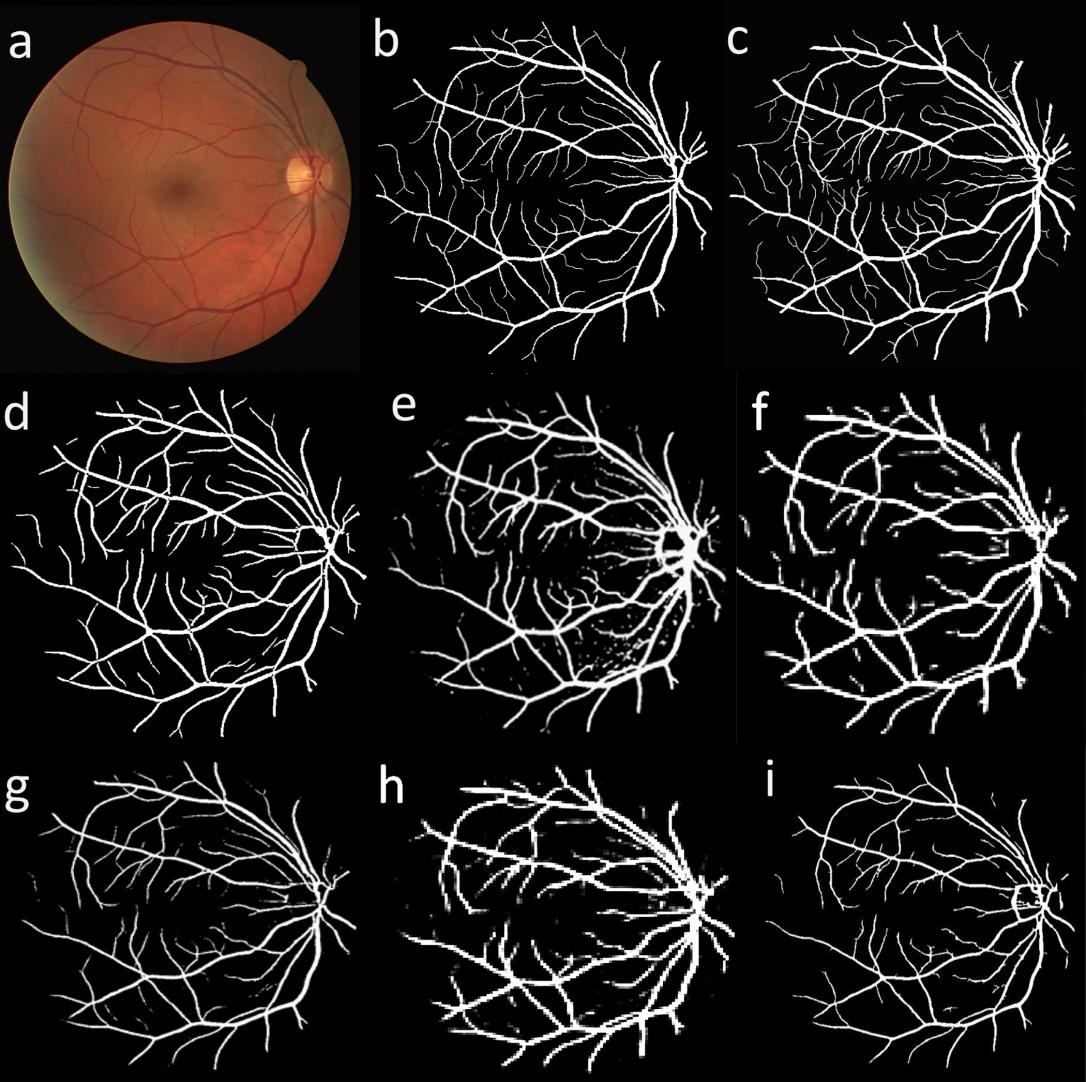
A visual comparison between different retinal vessel segmentation methods on a sample image from DRIVE dataset. (a) color fundus image, (b) manual segmentation by second observer, (c) manual segmentation by first observer, (d) proposed segmentation, (e) Wang et al. [28], (f) Marín et al. [24], (g) Aslani et al. [35], (h) Han et al. [74], (i) Maharjan et al. [76].

**Fig 14:**
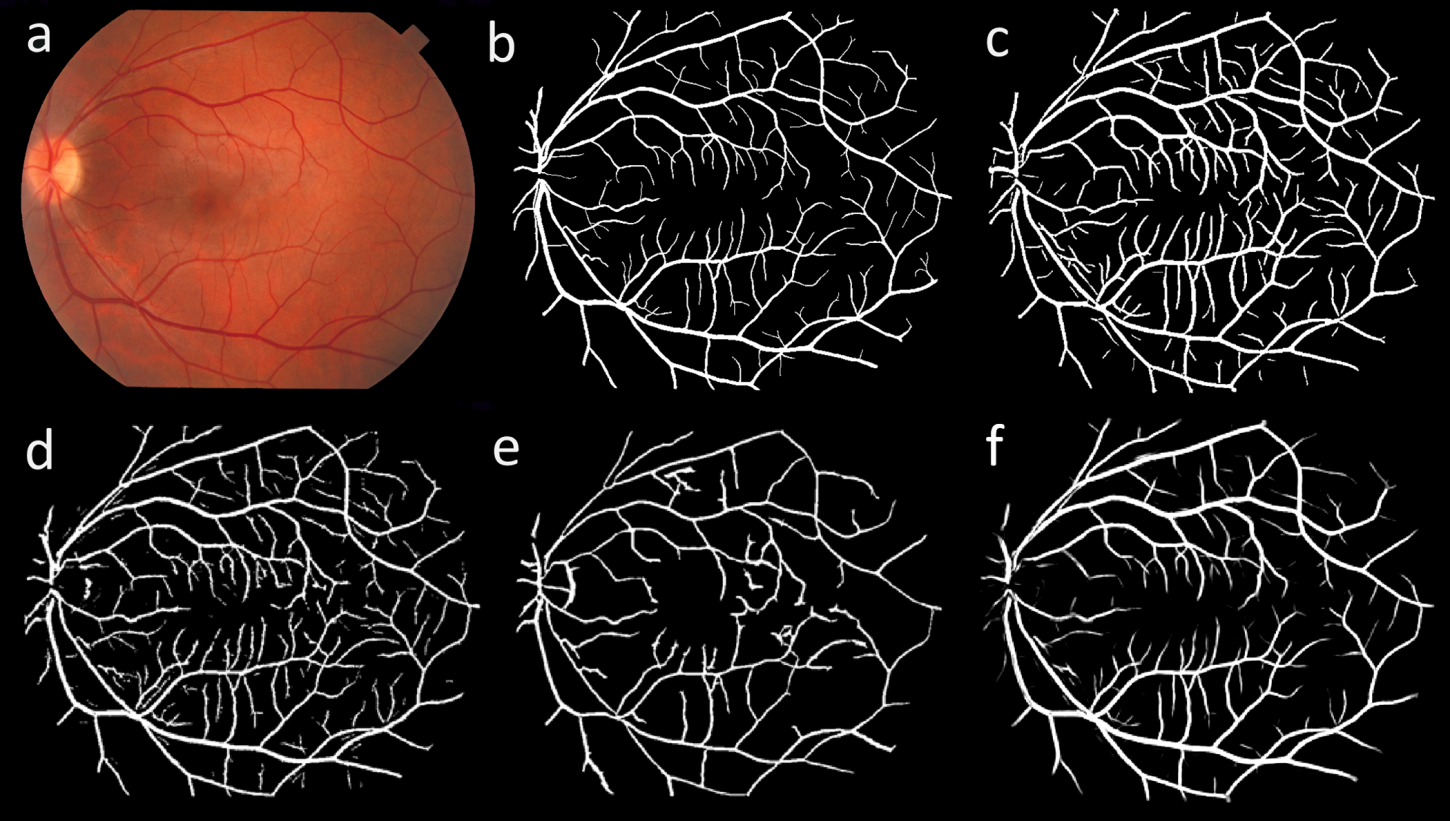
A visual comparison between different retinal vessel segmentation methods on a sample image from STARE dataset. (a) color fundus image, (b) manual segmentation by first observer, (c) proposed segmentation, (d) Peng et al. [75], (e) Hoover et al. [36] and (f) Soares et al. [15].

**Fig 15:**
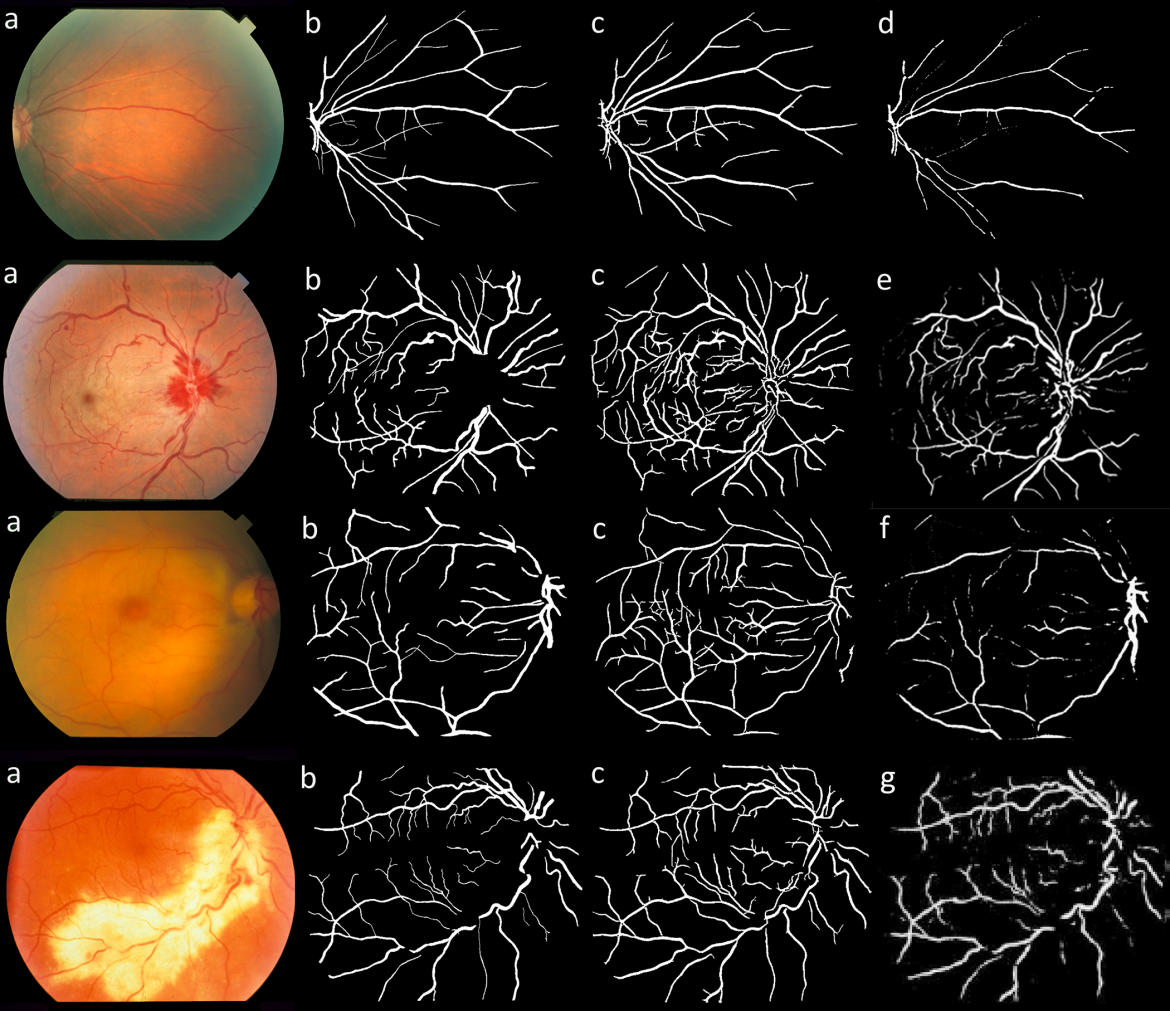
A visual comparison between different retina vessel segmentation methods on sample pathological images from STARE dataset. (a) color fundus image, (b) manual segmentation by first observer, (c) proposed segmentation, (d) Rodrigues et al. [77], (e) Aslani et al. [35], (f) Wang et al. [28], (g) Han et al. [74].

Figs 13 and 14 illustrate a visual comparison between the vessel segmentation performance of the proposed method and other state of the art methods for a sample image from DRIVE and STARE datasets, respectively. As seen, the proposed method was able to provide acceptable segmentation accuracy with low levels of noise and segmentation artifacts. Although the visual comparison is subjective, it can still be used to highlight the advantages and disadvantages of different segmentation approaches. As illustrated, thin vessels and the noise in the images can be considered as the main challenges in retinal vessel segmentation. Another advantage of the proposed method can be seen in pathological retinal images where vessel pixels can be easily identified as non-vessels, degrading the usefulness of the segmentation. As illustrated in Fig 15, the proposed method could provide less noisy segmentation compared to other methods from the literature on some sample pathological retinal images from STARE dataset.

## Conclusions

Vessel segmentation can be considered as an important step toward automated retina analysis tools. The segmented vessels can be used for advance retina image analysis such as computing the vessel tortuosity and diameter, differentiating arteries and veins along with measuring the arteriovenous ratio. Moreover, segmented vessels are routinely used as features in retinal disease classification systems that are used for identification of several systematic diseases such as stroke, hypertension or diabetes, to name a few. In this paper, a supervised retinal vessel segmentation algorithm based on matched filters and AdaBoost classifier is proposed. The image is enhanced using morphological operations, the contrast is increased utilizing CLAHE method and the image inhomogeneity is corrected by Retinex approach. Then, a combination of B-COSFIRE and Frangi matched filters are used to enhance the blood vessel network. From this enhanced image, using a sliding window, different pixel-wise statistical features are computed. Utilizing mRMR feature selection, a set of features were selected for use in an AdaBoost classifier while keeping the features as small as possible without sacrificing the segmentation accuracy. The proposed method could handle pathological retina images and produces good segmentation, especially in thinner vessels. The proposed segmentation method was validated on publicly accessible datasets using common validation metrics where the results in DRIVE (Sensitivity = 0.8726, Specificity = 0.9884), STARE (Sensitivity = 0.8085, Specificity = 0.9798) and CHASE_DB1 (Sensitivity = 0.8192, Specificity = 0.9591) datasets were shown to be comparable to all supervised and unsupervised methods from the literature.

## Algorithm availability

The data used to test the algorithm with source code and MATLAB implementation of algorithms used in Table 8 are included as supporting information. The DRIVE, STARE and CHASE_DB1 datasets are available at http://www.isi.uu.nl/Research/Databases/DRIVE/, http://www.ces.clemson.edu/~ahoover/stare/ and https://blogs.kingston.ac.uk/retinal/chasedb1/, respectively.

## Supporting information

S1 File Data used to test the algorithm. (RAR)

S1 Link MATLAB implementation of Bankhead et al. [12] available at http://petebankhead.github.io/ARIA/. (RAR)

S2 Link Source code of Sofka and Stewart [84] available at https://www.cs.rpi.edu/~sofka/vessels_exec.html. (RAR)

S3 Link MATLAB implementation of Soares et al. [15] available at https://sourceforge.net/projects/retinal/. (RAR)

S4 Link MATLAB implementation of Vlachos et al. [85] available at https://matlabfreecode.wordpress.com/2013/02/27/detection-of-vessels-in-eye-retina-using-line-tracking-algorithm-with-matlab-code/. (RAR)

S5 Link MATLAB implementation of Azzopardi et al. [17] available at http://www.mathworks.com/matlabcentral/fileexchange/37395. (RAR)

S6 Link MATLAB implementation of Nguyen et al. [86] available at http://people.eng.unimelb.edu.au/thivun/projects/retinal_segmentation/. (RAR)
